# Integrating artificial intelligence and multi-omics data for precision oncology in endometrial cancer: a narrative review

**DOI:** 10.1007/s10142-026-01957-2

**Published:** 2026-06-29

**Authors:** Oishee Mondal, Masuma Khatun, Ankita Lawarde, Sajitha Lulu S., Vino Sundararajan, Andres Salumets, Vijayachitra Modhukur

**Affiliations:** 1 Integrative Multiomics Lab, School of Bio Sciences and Technology, Vellore Institute of Technology, Vellore, 632014 Tamil Nadu India; 2 Department of Obstetrics and Gynecology, Helsinki University Hospital, University of Helsinki, Haartmaninkatu 8, Helsinki, 00290 Finland; 3 Department of Obstetrics and Gynecology, Institute of Clinical Medicine, University of Tartu, L. Puusepa 8, Tartu, 50406 Estonia; 4 Celvia CC AS, Tartu, Estonia; 5 Division of Obstetrics and Gynaecology, Department of Clinical Science, Intervention and Technology (CLINTEC), Karolinska Institutet, Karolinska University Hospital, Stockholm, Sweden

**Keywords:** Endometrial cancer, Artificial intelligence, Multi-omics, Spatial transcriptomics, Deep learning, Biomarker discovery, Precision oncology

## Abstract

Endometrial cancer (EC) is the most common gynaecological malignancy worldwide, yet the prognosis for advanced and recurrent disease remains poor, highlighting the need for improved diagnostic, prognostic, and therapeutic decision-making frameworks. Conventional approaches, including histopathology, imaging, and single-layer molecular profiling, provide essential clinical information but may not fully capture EC’s biological heterogeneity, especially within clinically challenging No Specific Molecular Profile (NSMP) and mismatch repair-deficient (MMRd) subgroups. Artificial intelligence (AI) and machine learning (ML) provide powerful approaches to analyse complex, high-dimensional datasets generated by multi-omics profiling, histopathology, imaging, and clinical records.

In this review, we synthesize the latest evidence on AI-driven multi-omics research in EC, encompassing genomics, transcriptomics, proteomics, metabolomics, epigenomics, single-cell profiling, and spatial transcriptomics. Unlike other reviews that focus solely on AI, omics, or imaging, we integrate molecular, imaging, histopathological, and computational perspectives to underscore their collective impact on precision oncology in EC. We subsequently explore applications in molecular subtyping, predicting survival and recurrence, modelling treatment responses, discovering immunotherapy biomarkers, and identifying drug targets. Public resources such as The Cancer Genome Atlas (TCGA), Clinical Proteomic Tumour Analysis Consortium (CPTAC), Gene Expression Omnibus (GEO), cBioPortal, Human Protein Atlas, GTEx, and UCSC Xena have enabled large-scale reproducible analyses. However, challenges such as cohort heterogeneity, batch effects, ethnic underrepresentation, missing annotations, and the need for external validation remain significant hurdles.

We then discuss the progression from conventional ML methods to deep learning architectures, including convolutional neural networks, transformers, graph neural networks, and multimodal fusion models applied to histopathological, radiological, and multi-omics data. Landmark models such as EndoNet, EndoRisk, and HECTOR illustrate the potential of AI-enabled approaches to support EC grading, molecular inference, lymph node metastasis prediction, and recurrence-risk stratification. Finally, we examine key translational barriers, including class imbalance, interpretability, data harmonization, regulatory requirements, and the implementation gap between high-performing retrospective models and routine clinical deployment. Ultimately, this review underscores how bridging these multi-modal computational approaches paves the way for precision oncology in EC.

**Supplementary Information:**

The online version contains supplementary material available at 10.1007/s10142-026-01957-2.

## Introduction

Endometrial cancer (EC) is the sixth most common gynaecological malignancy worldwide, with an estimated 420,000 new cases and 97,000 deaths reported in 2022 (Bray et al. 2024). EC remains the most common gynaecological malignancy in developed nations, with a rapidly rising incidence and mortality rate in China attributed to socioeconomic transitions and increasing metabolic risk factors. Between 2004 and 2021, there were 39,157 reported deaths, highlighting persistent rural-urban disparities in mortality and underscoring the urgent need for improved early detection and management strategies (Zhong et al. 2026). Most patients present with postmenopausal bleeding, enabling early diagnosis when EC is confined to the uterus, where surgical management yields a five-year survival rate of up to 95%. However, survival rates drop to approximately 18% once EC metastasizes beyond the uterus, and outcomes for advanced EC have remained largely unaltered despite multimodal therapies (Jo et al. 2025). These shortcomings highlight the necessity for enhanced molecular characterization and predictive tools to inform better diagnosis, prognosis, and treatment selection.

**Fig. 1:**
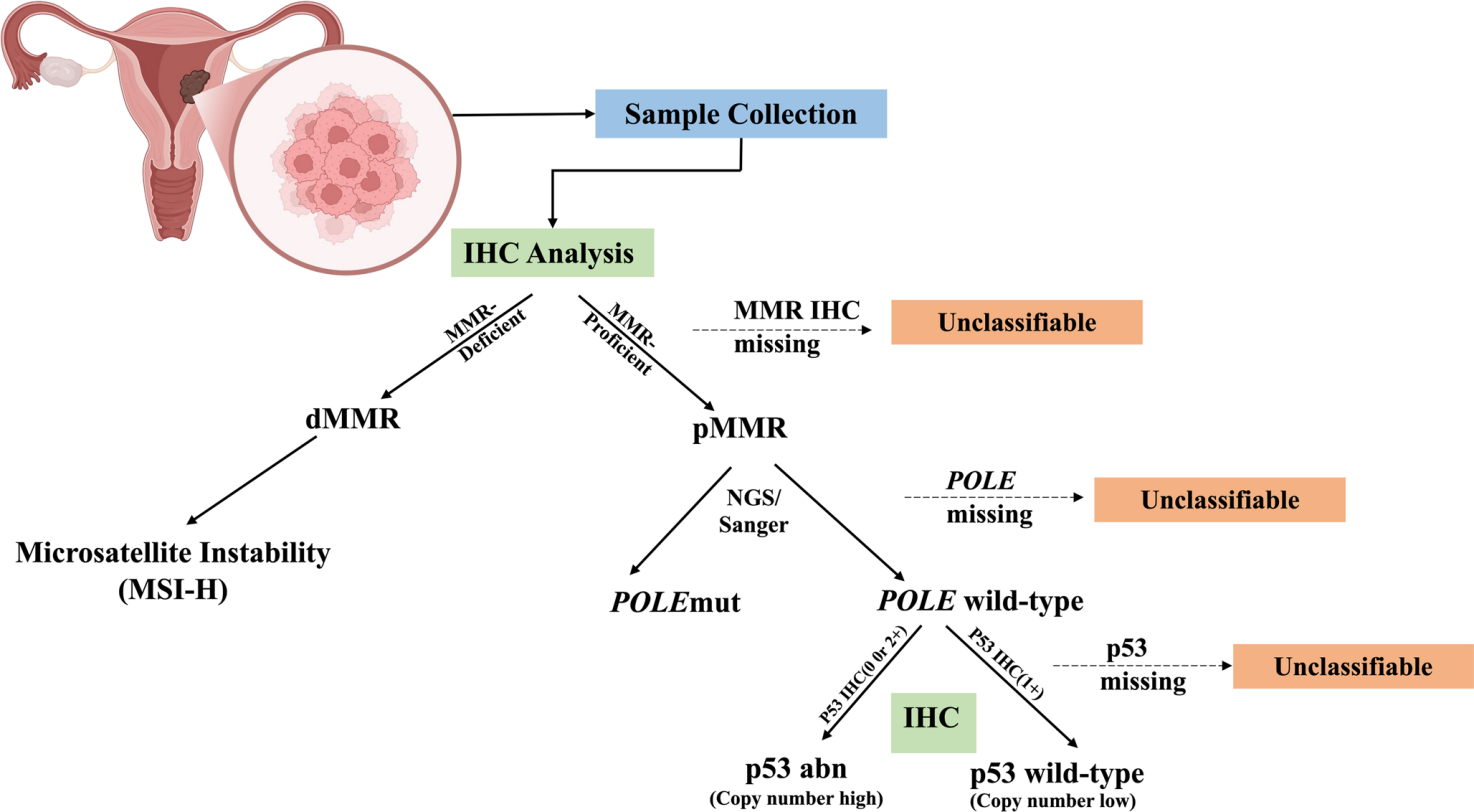
Overview of the ProMISE classifier and its translation of TCGA molecular taxonomy into clinical practice. Endometrial cancers are categorized into four molecular subtypes based on TCGA and the ProMisE algorithm: POLE-mutated, microsatellite instability or mismatch repair-deficient (MSI/MMRd), copy-number low (NSMP), and copy-number high characterized by TP53 abnormalities. These groups exhibit distinct genomic profiles, prognostic outcomes, and therapeutic implications.

The Cancer Genome Atlas (TCGA) molecular classification introduced in 2013 reshaped EC risk stratification by defining four biologically distinct subgroups (*POLE* mutated, MMRd/MSI-high, copy-number low (NSMP), and copy-number high (p53 abnormal), each with distinct prognostic trajectories (Getz et al. 2013). While *POLE*-mutated tumours show excellent outcomes, p53-abnormal tumours are associated with aggressive behaviour and poor survival (Khatun et al. 2025). Translation of the TCGA taxonomy into clinical practice through classifiers such as ProMisE (Fig. 1), based on MMR immunohistochemistry, *POLE* sequencing, and p53 status, has reshaped international guidelines, including FIGO 2023 and ESGO–ESTRO–ESP recommendations, thereby refining risk stratification and reducing overtreatment (Getz et al. 2013; de Biase et al. 2023; Ouh et al. 2024). The revised ESGO–ESTRO–ESP 2025 guidelines recommended assessment of estrogen receptor status within the NSMP subgroup. Furthermore, integrating myometrial invasion, lymphovascular space involvement, and tumour stage with molecular subtyping now defines risk groups and guides adjuvant therapy (Genovesi et al. 2026).

Despite these advances, substantial heterogeneity persists within molecular subgroups, particularly NSMP and MMRd tumours, and mechanisms of treatment resistance remain poorly understood (Joehlin-Price et al. 2021). Moreover, the widespread implementation of molecular classification is constrained by the cost, labour intensity, and scalability limitations associated with sequencing and immunohistochemistry (IHC) (Bogani et al. 2026). These challenges highlight the limitations of traditional single-layer omics approaches and emphasize the need for integrative strategies that can capture regulatory networks and functional pathways beyond mere genomic alterations. While single-omics analyses offer valuable insights, there remains an incomplete understanding of tumour biology. However, AI-driven multi-omics integration can combine genomic, transcriptomic, proteomic, imaging, and clinical data to uncover non-linear patterns linked to molecular subtype, lymph node metastasis, prognosis, and treatment response (Chen et al. 2020; Schoenaker et al. 2025; Jiang et al. 2025; Guo et al. 2026). Such integrative approaches have the potential to enhance risk stratification and support more precise clinical decision-making in EC.

The tumour microenvironment (TME) also profoundly influences EC progression, comprising a dynamic ecosystem of immune cells, stromal components, and extracellular matrix (Casas-Arozamena and Abal 2020). Notably, intercellular crosstalk within the TME plays a critical role in tumour evolution, immune evasion, and therapeutic resistance (Kim and Kim 2025). Capturing this complexity requires multi-omics approaches that integrate genomics, transcriptomics, proteomics, metabolomics, and epigenomics to characterise EC biology across molecular subtypes (Sibilio et al. 2025). However, the scale, dimensionality, and heterogeneity of these datasets pose challenges for conventional analytical methods, making artificial intelligence (AI) and machine learning (ML) increasingly important tools in EC research. The concurrent growth of multi-omics, radiomics, and digital pathology datasets further underscores the need for computational approaches capable of managing high-dimensional data and supporting biological interpretation (Esteva et al. 2019). In this direction, AI-assisted diagnostic systems using deep learning (DL) models are increasingly being explored for EC imaging modalities such as ultrasound, MRI, and CT (Tao et al. 2025). Beyond lesion detection, AI can also support image preprocessing and segmentation, thereby improving visualisation of endometrial architecture and localization of malignant features (Jiménez-Sánchez et al. 2023). While prior reviews have examined AI applications in EC or multi-omics landscapes in isolation, a critical synthesis that integrates these domains and specifically addresses how computational methods can resolve heterogeneity within existing molecular subtypes is lacking. This review addresses this gap by summarizing recent advances in AI-driven multi-omics analysis for EC and evaluating how integrative computational approaches support biomarker discovery, molecular classification, prognosis prediction, treatment-response modelling, and clinical translation. The review is organized as follows: In the Multi-Omics, Single-Cell/Spatial Profiling section, we discuss the major molecular layers and emerging single-cell and spatial approaches that define EC heterogeneity. In the Public Multimodal Data Repositories section, we summarize key data resources and their limitations for AI-enabled EC research. The Machine Learning in EC and Deep Learning Architecture and Application sections review computational methods for diagnosis, subtype classification, biomarker discovery, and outcome prediction, followed by landmark models and multimodal strategies that bridge histology, imaging, and genomics. In the Current Applications of Multi-Omics: From Diagnosis to Therapy section, we examine clinical applications including early detection, liquid biopsy, molecular subtype-stratified therapy-response prediction, and drug discovery. Finally, in the Practical Challenges, Future Directions, Ethical and Regulatory Considerations, and Review Limitations sections, we discuss the methodological, regulatory, and translational steps needed to move AI-enabled multi-omics from biomarker discovery Fig. 1: Overview of the ProMISE classifier and its translation of TCGA molecular taxonomy into clinical practice. Endometrial cancers are categorized into four molecular subtypes based on TCGA and the ProMisE algorithm: POLE-mutated, microsatellite instability or mismatch repair-deficient (MSI/MMRd), copy-number low (NSMP), and copy-number high characterized by TP53 abnormalities. These groups exhibit distinct genomic profiles, prognostic outcomes, and therapeutic implications toward clinically actionable precision oncology.

## Multi-omics, single-cell/spatial profiling

Advances in next-generation sequencing (NGS) have enabled systematic characterization of EC genomics (Kandoth et al. 2013). Exome sequencing has uncovered frequently altered genes such as *PTEN*,* ARID1A*, and *TP53* as significant determinants of EC pathogenesis, while transcriptomics [22] has revealed dynamic gene expression programs linked to prognosis and therapy response (Bhardwaj et al. 2022). For example, a transcriptomics analysis demonstrated *KIF11* upregulation as a poor prognostic indicator in EC, and knockout studies confirmed its role in tumour cell proliferation, migration, and invasion (Wang et al. 2025a, b, c, d). Proteomics bridged genotype to function by profiling protein abundance and post-translational modifications, and metabolomics provided real-time insights into tumour metabolic states (Yasar et al. 2024). For instance, dysregulation of lipid metabolism and glycolysis is a hallmark of EC, with altered metabolites such as acetylcholine, 3-hydroxybutyric acid, homocysteine, and lactic acid (Raffone et al. 2020). Epigenomic alterations further illuminated early carcinogenic events and immune responsiveness (Zhao et al. 2025). Driven by these advances, an integrated proteomics and whole-exome sequencing study revealed cabozantinib-associated immune signatures (increased plasma HO-1, reduced VEGFR2, and IL-12), which were associated with the clinical outcomes (Roudko et al. 2025).

Revolutionary techniques such as single-cell RNA sequencing (scRNA-seq) and spatial transcriptomics (ST) have since transformed EC research by resolving intra-tumour heterogeneity obscured in bulk analyses, identifying rare malignant subpopulations, and mapping immune–tumour interactions within intact tissue architecture (Mondal et al. 2025). As tumour cell states evolve dynamically during progression, signal averaging in bulk transcriptomics often masks rare but clinically relevant subclonal populations (Zhang et al. 2021a). ScRNA-seq overcomes this limitation by profiling gene expression at single-cell resolution, enabling detailed characterization of tumour, immune, and stromal compartments, identification of drug-sensitive cell subsets, and discovery of cluster-specific therapeutic targets (Chen et al. 2024). However, scRNA-seq lacks spatial context, which ST restores by mapping gene expression within intact tissue architecture (Li et al. 2025b). Spatial profiling of the TME integrates molecular signals with histology, improving prediction of disease aggressiveness and treatment response (Cilento et al. 2024). Notably, ST-based studies of anti–PD-1 therapy have shown that responders exhibit active CD8⁺ cytotoxic and regulatory T-cell states and enhanced ligand–receptor communication networks, whereas non-responders display suppressed immune activity (Chen et al. 2024).

AI has the potential to strengthen the link between spatial transcriptomics and digital pathology by integrating spatial gene-expression profiles with histological features from H&E-stained tissue sections (Guo et al. 2026). Deep learning and graph neural networks (GNNs) can relate spatial molecular signals to local tissue architecture and tumour–immune neighbourhoods, supporting detailed characterization of the tumour microenvironment (Zhang et al. 2025a). However, the high cost and technical complexity of spatial transcriptomics limit the formation of large patient cohorts required for reliable biomarker discovery. Emerging AI-driven approaches, such as Path2Space, address this limitation by predicting spatial gene-expression patterns directly from H&E slides, thereby expanding the potential use of spatial information in larger cohorts (Shulman et al. 2024). Table 1 summarizes major multi-omics layers in EC, highlighting their biological rationale, clinical utility, strengths, limitations, and detection feasibility.

The value of multi-omics integration lies in connecting genotype, regulation, function, and phenotype. Genomic alterations such as *PTEN*, *TP53*, *POLE*, and *CTNNB1* mutations can influence transcriptional programmes, protein signalling, metabolic states, and epigenetic regulation (Molefi et al. 2025). Each omics layer contributes distinct but complementary information: genomics identifies stable driver alterations, transcriptomics captures dynamic gene-expression changes, proteomics reflects functional protein-level regulation, metabolomics provides readouts of tumour metabolic activity, and epigenomics reveals regulatory mechanisms that may influence tumour behaviour and therapy response (Boroń et al. 2022). Together, these layers offer a more comprehensive framework for biomarker discovery, molecular classification, prognosis prediction, and treatment-response prediction in EC.

**Table 1:** Integrated multi-omics landscape of endometrial cancer: biological rationale and clinical utility

Omics layer	Representative biomarkers	Core pathways	Biological and clinical utility	Strength	Clinical detection feasibility	Limitations	References
Genomics	*POLE*,* TP53*,* PTEN*,* PIK3CA*,* ARID1A*,* CTNNB1*,* KRAS*	PI3K/AKT/mTOR, WNT/β-catenin, DDR, MAPK	Molecular subtype, prognosis, therapy selection	Stable, reproducible, clinically established	High–standardized NGS/PCR assays are available	Limited functional resolution; spatial heterogeneity	(Ruz-Caracuel et al. 2021; Molefi et al. 2025)
Transcriptomics	*ECT2*,* WFS1*	Cell cycle, EMT, glycolysis, immune modulation	Risk stratification, predicts recurrence	Captures dynamic tumour states	Moderate – feasible via RNA-seq/RT-qPCR, limited by RNA instability	Sensitive to sampling, ischemia	(Li et al. 2024b; Wu et al. 2024)
Proteomics	HE4, CPN10	JAK/STAT, proteolysis, stress response	Diagnosis, treatment resistance prediction	Associated with phenotype	Moderate – Some markers are clinically validated (HE4), others remain exploratory	Limited clinical assays; protein instability	(Serambeque et al. 2024)
Metabolomics	3-Hydroxybutyrate, Inosine	β-oxidation, TCA cycle, purine metabolism	Early detection and monitoring	Early disease signal; biofluid-based	Low – limited standardization	Diet and microbiome influence	(Albertí-Valls et al. 2023)
Epigenomics	*hMLH1*,* RASSF1A*,* CDH13* (methylation)	MSI, chromatin remodelling, cell cycle control	Non-invasive diagnosis and therapy response	Detectable in minimally invasive samples	High – compatible with liquid biopsy and methylation- specific PCR assays	Cell-type dependence	(Piergentili et al. 2025)

## Public multimodal data repositories

**Fig. 2:**
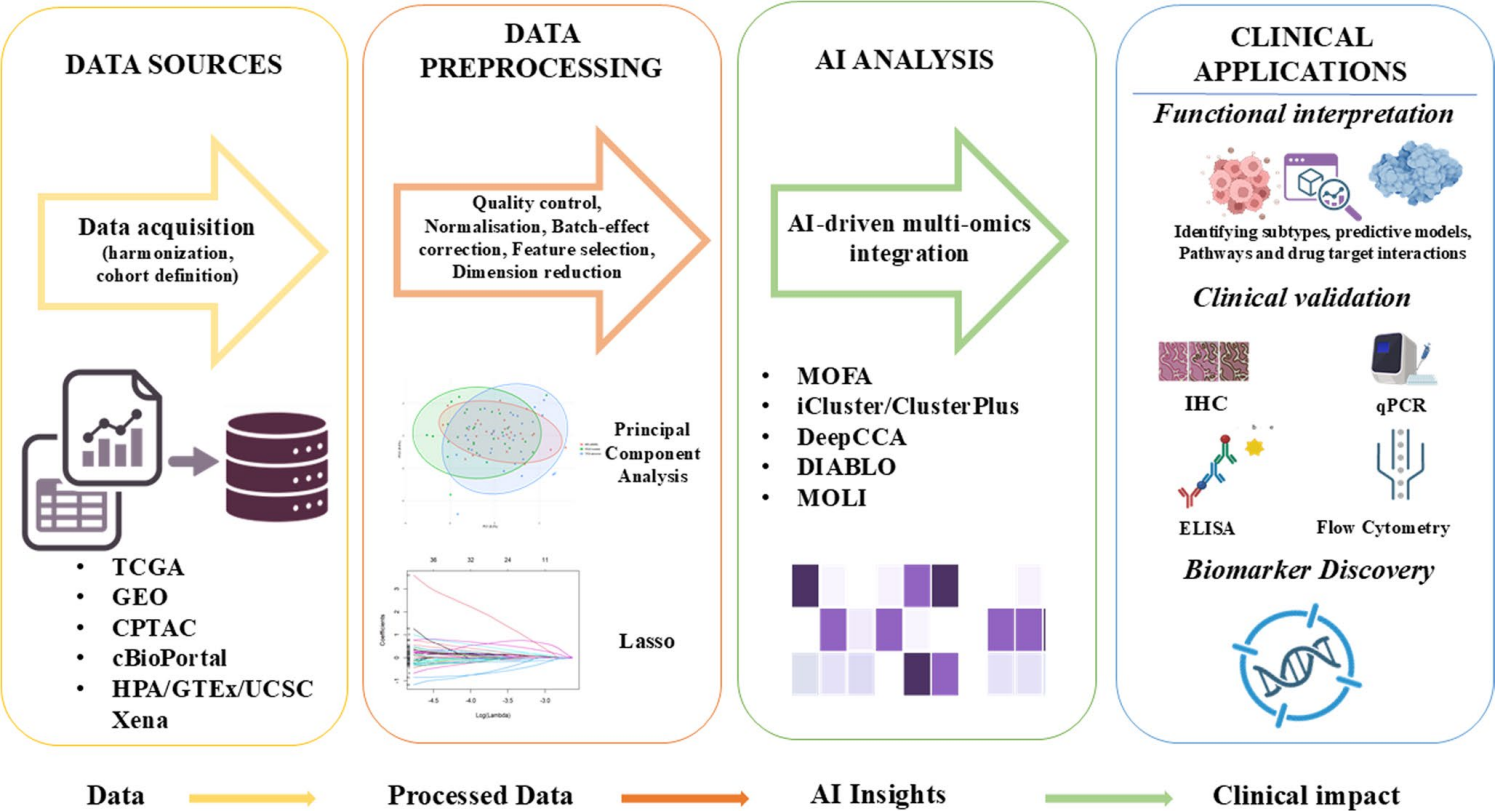
Public and Multimodal Data Sources to Clinical Impact Pipeline for Endometrial Cancer precision Oncology. Multi-omics datasets from TCGA, CPTAC, and GEO undergo preprocessing (quality control, normalisation, PCA, and Lasso regression). Standardised data layers are fused using advanced AI and deep learning architectures (MOFA, DeepCCA, GNNs, and transformers). These integrated models enable downstream functional interpretation, including molecular subtyping, pathway crosstalk analysis, and drug target identification, which are experimentally validated (via IHC, qPCR, ELISA, and flow cytometry) for actionable biomarker discovery.

Ethical and logistical constraints limit direct access to patient cohorts; curated public repositories have become essential for reproducible and scalable EC research (Fig. 2). Large-scale initiatives such as TCGA, Gene Expression Omnibus (GEO), and Clinical Proteomic Tumour Analysis Consortium (CPTAC) provide harmonized genomic, transcriptomic, proteomic, and imaging data. TCGA, which includes the uterine corpus endometrial carcinoma (TCGA-UCEC) (2025), and comprises over 11,000 samples across cancer types, has enabled integrative studies identifying clinically relevant biomarkers, including cancer-testis antigens such as TTK, which is associated with chemoresistance, epithelial-mesenchymal transition, and poor prognosis (Miao et al. 2023).

Complementary proteomic insights from CPTAC link protein expression with transcriptomic and genomic alterations, revealing adverse prognostic markers such as *CCDC138* and validating targets like TTK at the protein level (Wang et al. 2025a, b, c, d). Transcriptomic and epigenomic resources from GEO (Barrett et al. 2005) and ArrayExpress (Parkinson et al. 2006)together with integrative platforms such as cBioPortal (Cerami et al. 2012), Human Protein Atlas, GTEx, and UCSC Xena further expand analytical capacity by enabling tumour–normal comparisons, immune profiling, and multi-omics validation using pre-processed, standardized datasets (Austin et al. 2023). Collectively, these repositories underpin AI-enabled multi-omics integration, accelerating translation from molecular discovery to precision oncology in EC. An overview of publicly available EC databases and their applications in ML-based studies is provided in Supplementary Table S1.

However, public databases also have important limitations that can affect the robustness and generalizability of AI-based investigations. One major concern is demographic and ethnic imbalance, as many available EC datasets are derived predominantly from Western cohorts, with limited representation of non-Western populations. For example, the TCGA-UCEC cohort retrieved from the Genomic Data Commons (GDC) portal primarily includes samples from the United States (*n* = 293), with smaller representation from Canada (*n* = 36), Russia (*n* = 2), and Ukraine (*n* = 2), while demographic information is unavailable for several samples (2025). In addition to population imbalance, technical and clinical heterogeneity across repositories can introduce analytical bias. Differences in cohort size, tissue collection, sequencing platforms, coverage depth, preprocessing pipelines, clinical definitions, follow-up duration, and annotation quality may reduce reproducibility and limit model transferability across datasets. To address these challenges, multi-database AI studies require standardized integration workflows. Such workflows should include dataset selection based on a clearly defined clinical endpoint, raw-data quality control, harmonization of clinical annotations, platform-specific normalization, batch-effect correction, missing-data handling, feature matching across omics layers, and cohort-level rather than random train–test splitting to reduce data leakage (Cai et al. 2022; Baião et al. 2025). External validation in independent cohorts is essential to confirm that selected biomarkers and model predictions reflect tumour biology rather than dataset- or centre-specific technical effects (Cai et al. 2022; Austin et al. 2023; Baião et al. 2025). Finally, biological interpretation through pathway analysis or explainable AI methods can help distinguish clinically meaningful tumour-specific signals from technical noise (Cai et al. 2022; Baião et al. 2025).

## Machine learning in EC: from diagnosis to prediction

Machine learning (ML), including deep learning (DL), is a core component of AI that enables data-driven modelling for classification and prediction (Bhardwaj et al. 2022). By learning complex, often non-linear relationships, ML is particularly well-suited to high-dimensional biomedical data. In EC, ML applications have expanded from diagnosis and staging to recurrence prediction, molecular subtyping, and survival modelling (Vela Moreno et al. 2026). One clinically critical application is the pre-treatment assessment of lymph node metastasis (LNM), which strongly influences prognosis and therapeutic decisions. Radiomics-based ML models using multiparametric MRI (T2WI, DWI, DCE) have demonstrated robust performance, achieving AUCs up to 0.900 in internal testing and 0.858 in external validation, highlighting generalizability across centres (Tao et al. 2025). Beyond imaging, ML has also enabled the identification of circulating biomarkers for EC detection and stratification. Gradient Boosting models such as LightGBM and XGBoost have successfully discriminated EC cases from controls using serum metabolomics, with SHAP analysis improving model interpretability (Clin et al. 2025). Table 2 summarises key biomarker-driver ML studies in EC to illustrate the diversity of ML applications across omics, imaging, and clinical datasets. The evaluation of prediction performance of ML and DL-powered models relies on various computed measures from the confusion matrix, such as TP (true positive), TN (true negative), FP (false positive), and FN (false negative). Commonly used metrics include accuracy, recall, precision, and F1 score (Rainio et al. 2024). A detailed summary of these evaluation metrics is provided in Supplementary Table S2.

**Table 2:** Biomarker-Based Machine Learning Approaches for Endometrial Cancer Classification and Prognosis

Biomarker gene	Type (mRNA/ lncRNA/ protein)	Input data type (Omics/ imaging/ clinical)	Sample size (internal/external)	Algorithm/ model	Classification purpose (subtypes/ prognosis/ risk groups)	Reference
LRPPRC	mRNA	Transcriptomics	Internal: TCGA-UCEC (*n* = 554 EC cases/35 controls) External: TCGA-CESC (*n* = 168 cases), TCGA-BRCA (*n* = 1057 cases)	LASSO-Cox	Risk stratification	(Xiong et al. 2023)
*POLE*mut, MMRd, p53abn, NSMP	Protein + DNA mutations	Imaging (H&E WSIs)	Internal: *n* = 393 EC cases (*n* = 15328 WSIs); External: Independent cohorts (*n* = 35; *n* = 83)	SRResGAN + MedSAM + ResNet-101 + LSTM (DL pipeline)	Molecular subtype + prognosis prediction	(Qi et al. 2025)
CVF panel + plasma panel	Protein	Proteomics (SWATH-MS)	118 (EC,65 controls; 80:20 cv split)	RF + Boruta + LR	EC detection (overall & stage I)	(Njoku et al. 2024)
8-protein signature	Protein	CPTAC proteomics	CPTAC EC cohort (*n* = 95;83 endometrioid, 12 serous; 70:30 split)	LASSO LR + SMOTE + SHAP/LIME	EC subtypes & TMB classification	(Luong et al. 2025)
Stemness mRNAs (mRNAsi)	mRNA	Transcriptomics	Internal: TCGA-UCEC (Train and test split at 2:1 ratio) External: In-house dataset (*n* = 24)	LASSO + RF+ Cox	Stemness subtype + prognosis	(Pang et al. 2024)
Proline betaine and LPE	Metabolites	UHPLC-HRMS metabolomics	Internal: 3 gynaecological cancers *n* = 42 (EC = 9) + 57 controls External: none	OPLS-DA, Lasso, Bagging, RF, Boosting, SVM	Subtype classification	(Cha et al. 2025)
PARD6G-AS1 (hypomethylation), CD44 (overexpression)	Methylation + mRNA	TCGA multi-omics	Omics data: TCGA-UCEC External: In-house dataset (*n* = 16)	Decision Tree, RF	Recurrence prediction	(Hong et al. 2025)

### Supervised ML models

Supervised ML algorithms infer predictive relationships from labelled data and are broadly categorized into classification and regression tasks (Yaqoob et al. 2023). Support Vector Machine (SVM) is widely used in EC for gene expression-based subtype classification and recurrence prediction due to its ability to model non-linear decision boundaries (Dong et al. 2024). Decision Trees offer interpretability; however, they are prone to overfitting (Yaqoob et al. 2023). This limitation is mitigated by ensemble methods, particularly Random Forest (RF), which facilitates bootstrap aggregation of multiple trees (Samarasam and Justin 2025). RFs are widely used for EC outcome prediction and feature ranking, particularly in radiomics-based ML models (Coada et al. 2023). Comparative studies have shown RFs to outperform other supervised methods in predicting myometrial invasion and metastasis (Chen et al. 2025). Other classifiers include K-Nearest Neighbour (KNN) for pattern recognition tasks (Dong et al. 2024)and Naïve Bayes, for predicting lymph node involvement using histopathological features (Günakan et al. 2019). Regularized regression techniques are fundamental in omics-driven EC research. Least Absolute Shrinkage and Selection Operator LASSO (L1 penalty) enables simultaneous feature selection and prediction (Tibshirani 1996), Ridge regression (L2 penalty) stabilizes coefficient estimates (Hernández-Lemus and Ochoa 2024), and Elastic Net combines both penalties to balance sparsity and robustness. These approaches are routinely used for biomarker discovery and noise reduction in EC datasets (Chen et al. 2022).

### Unsupervised clustering

Clustering algorithms like k-means are applied in EC Imaging and Molecular data to stratify tumours based on similarity (Bhardwaj et al. 2022; Yang et al. 2025). Non-negative Matrix Factorization (NMF) is particularly suited for molecular subtyping in EC due to its parts-based non-negative representation (Zhou et al. 2025). Hierarchical clustering provides multi-scale insights through dendrograms (Moufarrij et al. 2025), while Consensus clustering (e.g., ConsensusClusterPlus)(Wilkerson and Hayes 2010) is widely exploited on transcriptomics and multi-omics datasets to stratify patients into reproducible molecular subtypes (Liu et al. 2024; Yin and Luo 2025). Given the high dimensionality and noise inherent in biological data, dimensionality reduction techniques are routinely employed. Principal Component Analysis (PCA) captures dominant linear variance (Švecová et al. 2025), whereas t-distributed stochastic neighbour embedding (t-SNE)(Yao et al. 2024) and Uniform manifold approximation and projection (UMAP) preserves non-linear local (and, for UMAP, global) structure (Xu et al. 2024). Anomaly detection methods further aid in identifying outliers (Torabi et al. 2023).

### Feature selection techniques

Feature selection is crucial in EC studies since multi-omics and radiomic datasets contain thousands of variables relative to limited patient cohorts, increasing overfitting risk and unstable biomarker discovery. In multi-omics studies, these methods are frequently combined with downstream survival modelling or deep learning pipelines to reduce dimensionality while preserving biologically relevant signals. These methods are broadly categorized into filter, wrapper, and embedded approaches (Al-Tashi et al. 2020). Filter methods (e.g., chi-square, correlation-based selection) are computationally efficient and well-suited to omics-scale data (Cheng et al. 2021). Wrapper methods evaluate feature subsets using predictive models but are prone to overfitting. Embedded methods integrate feature selection directly into model training, balancing performance and efficiency (Pudjihartono et al. 2022). In EC studies, embedded approaches such as RF-based importance ranking and regularization techniques (LASSO, Elastic Net) are most widely used for gene, protein, and radiomic feature selection (Wu et al. 2023). Feature selection methods used in EC and their advantages, limitations, and applications are summarised in Supplementary Table S3.

### Multi-omics integration and ML

Multi-omics integration combines genomics, transcriptomics, epigenomics, proteomics, and metabolomics to provide a systems-level view of EC biology. Integration strategies include vertical integration, measuring multiple omics layers from the same samples, and horizontal integration, comparing the same omics layer across different samples or cohorts (Hodeify 2026). Analytically, these approaches can be classified as early or feature-level integration, intermediate or model-level integration, and late or decision-level integration (Picard et al. 2021).

It has to be noted that early, intermediate, and late integration strategies differ substantially in their computational requirements, interpretability, and suitability for clinical translation (Cai et al. 2022; Hernández-Lemus and Ochoa 2024; Baião et al. 2025). For instance, early fusion is relatively simple because features from different omics layers are combined before model training, but it can be affected by high dimensionality, missing values, noise accumulation, and dominance of data-rich modalities (Cai et al. 2022; Baião et al. 2025). However, intermediate fusion learns shared latent representations across modalities and may be particularly informative for EC molecular subtyping and risk stratification because it can preserve cross-layer interactions among genomic alterations, transcriptional programmes, protein signalling, and clinical phenotypes (Cai et al. 2022; Baião et al. 2025). However, this approach requires careful normalization, robust missing-data handling, and greater computational resources. Late fusion combines outputs from independently trained modality-specific models and is therefore more practical when some data types are unavailable, as is common in clinical settings; however, biological interactions between omics layers may be less directly captured (Cai et al. 2022; Ghaleb et al. 2026). Therefore, in EC research, intermediate fusion may be useful for mechanistic discovery and refinement of heterogeneous NSMP or MMRd subgroups, whereas late fusion may be more feasible for clinically deployable decision-support systems.

**Fig. 3:**
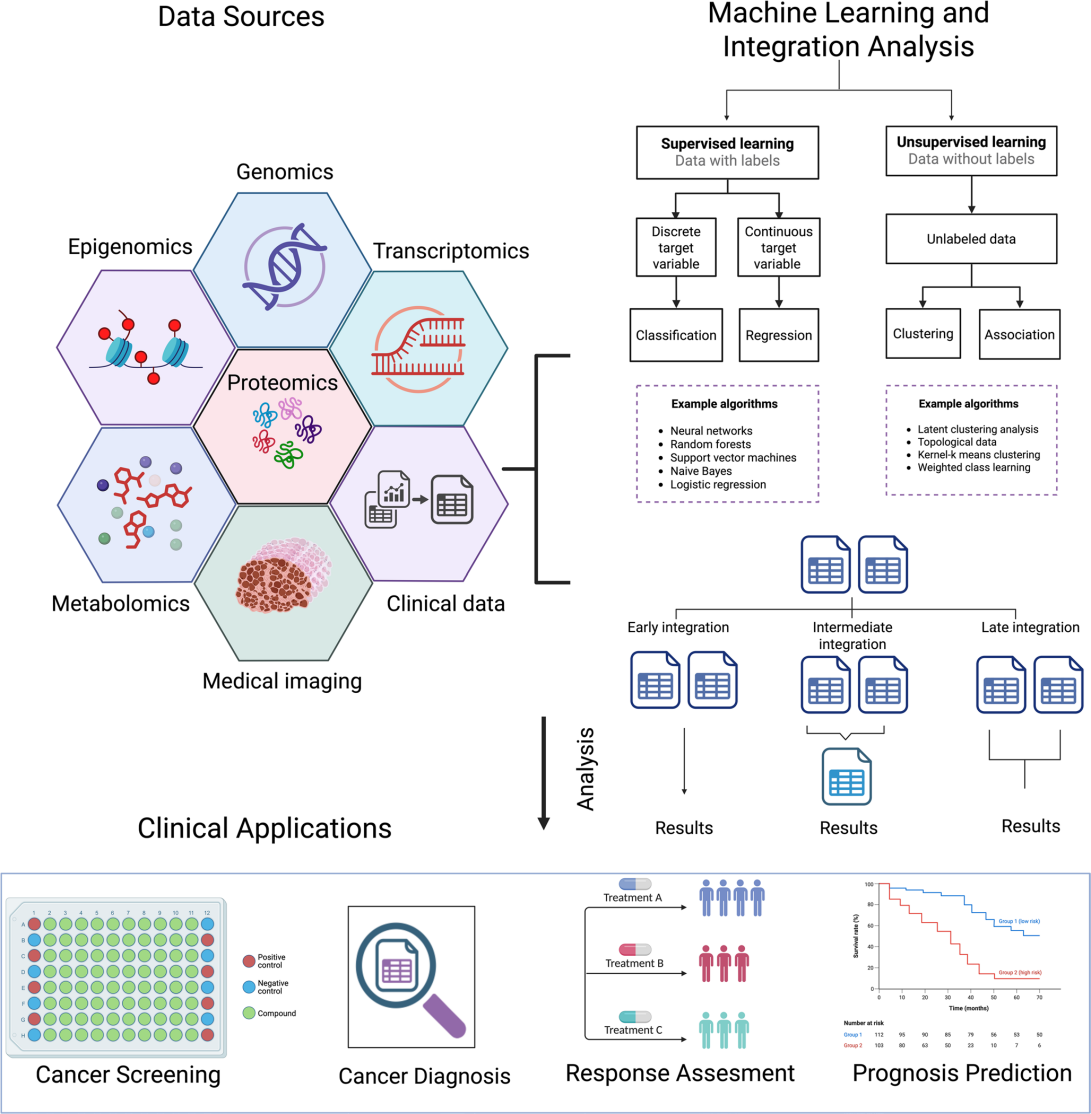
Multi-Omics Architecture of Endometrial Cancer. Genomic, transcriptomic, proteomic, metabolomic, and epigenomic datasets are processed through preprocessing, feature selection, and dimensionality reduction before integration using computational methods. Machine learning models then identify biomarkers and support outcome prediction and precision oncology strategies

ML and AI methods, including matrix factorization, network-based modelling, graph-based learning, and deep learning architectures, are central to these integration frameworks (Cai et al. 2022; Baião et al. 2025). These approaches support molecular subtyping, biomarker discovery, recurrence prediction, therapeutic-response modelling, and precision oncology decision-making. Importantly, successful multi-omics integration requires not only predictive accuracy but also biological interpretability, external validation, and careful control of technical bias (Baião et al. 2025). Figure 3 illustrates the multi-omics architecture of EC and its linkage to clinical decision-making, while commonly used ML-based multi-omics integration tools are summarised in Supplementary Table S4.

## DL architecture and application

**Fig. 4:**
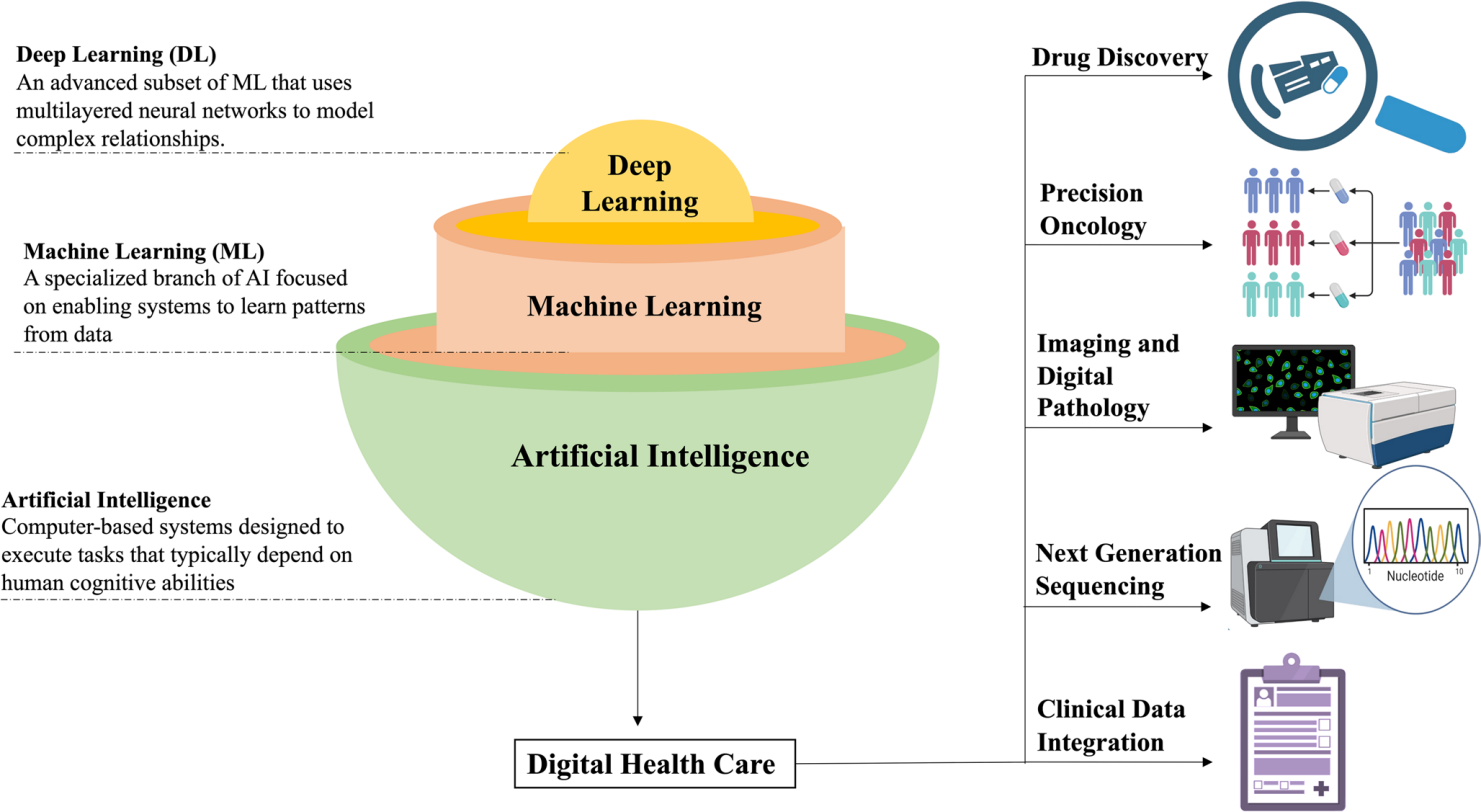
Artificial Intelligence, Machine Learning, and Deep Learning Frameworks for Endometrial Cancer Data: Artificial intelligence encompasses machine learning and deep learning algorithms that enable analysis of complex biomedical datasets, including genomic, imaging, and clinical data. These approaches support biomarker discovery, digital pathology, drug discovery, and precision oncology.

Deep learning (DL) is an extension of ML designed to analyse complex and high-dimensional biomedical datasets (Esteva et al. 2019). DL incorporates multi-layer neural networks inspired by brain architecture and enables end-to-end learning, directly mapping raw inputs (e.g., images, sequences, omics data) to predictions while reducing human bias (Hodeify 2026). Figure 4illustrates the relationship between AI, ML, and DL in EC research.

Histopathological assessment and grading of EC are challenging due to intra- and interobserver variability, overlapping morphological features, and intrinsic tumour heterogeneity. DL approaches may enhance automated and reproducible histopathological analysis. Unlike conventional ML algorithms relying on manually engineered features, DL models learn hierarchical spatial and morphological representations directly from WSIs (Tran et al. 2021; Song et al. 2022). In EC, CNN-based and multi-resolution DL approaches, including Panoptes and im4MEC models, show promise in predicting histological and molecular subtypes, such as *POLE* mutations, MMR deficiency, and TP53 alterations (Hong et al. 2021; Fremond et al. 2023). Beyond image analysis, DL is increasingly used for high-dimensional and heterogeneous datasets, integrating imaging, molecular, and clinical features to identify complex patterns relevant to diagnosis, prognosis, and treatment response (Tran et al. 2021). For example, integrative multi-omics analysis of EC tissues, urine, and uterine brush samples revealed disruptions in amino acid and nucleotide metabolism, identifying consistent metabolic signatures that may aid early EC detection (Yi et al. 2022).

### DL Architectures

DL architectures have evolved to address different biomedical data structures **(**Table 3**).** The earliest model, the multilayer perceptron (MLP), utilises fully connected layers and is effective for structured tabular data; however, it cannot capture spatial, temporal, or relational patterns (Alharbi and Vakanski 2023). This limitation led to specialized architectures such as convolutional neural networks (CNNs), recurrent neural networks (RNNs), and graph neural networks (GNNs) (Oo et al. 2025). CNNs are the backbone of computer vision, using convolution and weight sharing to learn hierarchical spatial features efficiently. In EC research, CNNs have shown strong performance in radiologic and histopathological analysis (Sarvamangala and Kulkarni 2022; Sharma et al. 2024). For example, a ResNet-101 model achieved high diagnostic (AUC 0.918) and recurrence prediction accuracy (AUC 0.926) from MRI data, supporting image-based risk stratification, although these models require large, annotated datasets and high computational resources (Qi 2024). In contrast, RNNs are designed to model sequential and temporal dependencies through recurrent connections, enabling superior performance on ordered data. An RNN outperformed SVMs in EC detection from pap-smear images, but standard RNNs suffer from gradient instability, which is mitigated by advanced variants such as long short-term memory (LSTM) and gated recurrent unit (GRU) networks (Reddy and Puviarasi 2022). Complementing CNNs and RNNs, GNNs are optimised for relational data by aggregating information from connected nodes to generate context-aware representations (Maurya et al. 2023). An explainable GNN framework integrating RNA-sequencing and DNA methylation data achieved an AUC of 0.99 for microsatellite instability prediction in EC and identified clinically relevant molecular markers. Together, these architectures demonstrate how DL models are tailored to spatial, sequential, and relational data to enable accurate and clinically meaningful prediction (Cao et al. 2023).

**Table 3:** Application of Deep Learning Architectures in Endometrial Cancer Diagnosis and Prognosis

Sample type	Algorithms	Sample size (internal/external)	Performance	Key features	References
EC Hysteroscopic images	CNN (VGGNet-16)	454 participants Train/test → 6478/250 Images	Accuracy 80.8, Specificity 96.0%	Automated lesion classification	(Zhang et al. 2021b)
MRI	Radiomics + RF	Train→ SMOTE-balanced (128:128) Test→ Validation set 1(*n* = 351 EC) & set 2 (271 EC)	AUCs 0.814–0.842, accuracy ~ 90%	Improved pelvic LNM detection	(Yan et al. 2021)
Histopathology	ECgMLP (gated MLP)	Total →26,380 images train/test/validation→70:20:10 ratio	Accuracy ~ 99.26% and AUC 1.00.	Multi-class diagnosis of EC	(Sheakh et al. 2025)
H&E WSI	HIENET (CNN + attention architecture)	Internal→3300 H&E patches (~ 500 patients) External → 200 H&E patches (~ 200 patients)	AUC ~ 0.96; Sensitivity 81%; External accuracy 84.5%	Pathologist-level EC diagnostic performance	(Sun et al. 2020)
Cytology slides	U-Net + DenseNet201 (classification)	113 samples (42 EC + 71 benign) Train/test/external→26,880/11,520/600 malignant patches	Accuracy 93.5%	Recognition of endometrial cell clumps (ECCs) in cytology	(Li et al. 2022)
Hysteroscopic images	Xception, EfficientNetB0, and MobileNetV2 + temporal continuity analysis	Only internal validation 177 videos (~ 411,800 frames; 4-fold train/evaluation CV)	Accuracy 90.29%; sensitivity 91.66%, specificity 89.36%	Video-based EC detection	(Takahashi et al. 2021)
TCGA (RNA-seq)	Graph Convolutional Network	Train/test: 186 / 46 graphs	Accuracy 99%, 23 biomarkers identified	Biomarker discovery and molecular typing	(Wu et al. 2022)

## AI Application in EC pathology and molecular profiling

AI-based pathology and multi-omics approaches offer opportunities to further refine EC molecular subgroups, particularly NSMP and MMRd tumours, where clinicopathological behaviour and treatment response can vary (Darbandsari et al. 2024). These approaches can identify morphological, spatial, and molecular patterns that may not be fully captured by conventional clinicopathological markers (Volinsky-Fremond et al. 2024). In NSMP tumours, attention-based multiple instance learning and other whole-slide image approaches can extract quantitative histological features from routine H&E slides and relate them to molecular alterations such as *CTNNB1* mutation, Wnt/β-catenin pathway activity, hormone-receptor status, and immune contexture (Qi et al. 2025). When integrated with clinical and molecular data, these approaches may support improved prognostic assessment and help identify patients who require closer monitoring or additional therapeutic consideration (Li et al. 2023).

In the case of MMRd tumours, AI-based integration of spatial transcriptomics, digital pathology, and immune profiling may improve understanding of heterogeneous immunotherapy responses (Guo et al. 2024). Graph-based and spatially aware deep learning models can represent tumour, immune, and stromal cells as interacting neighbourhoods, allowing analysis of tumour–immune architecture, cytotoxic T-cell infiltration, immune exclusion, and extracellular-matrix-associated barriers (Jiménez-Sánchez et al. 2022). Such approaches may help distinguish immune-active from immune-suppressed MMRd tumours and identify features associated with response or resistance to immune checkpoint inhibition. Overall, AI-driven integration of histology, spatial organization, and molecular data may support more refined risk stratification within NSMP and MMRd subgroups, although prospective validation and standardized clinical workflows are still required before routine implementation (Wu et al. 2025; Hollenberg et al. 2026).

### Case studies and landmark models

Several landmark models illustrate AI applications in EC diagnosis, molecular inference, and risk stratification. **EndoNet**, a transformer-based deep learning model, was developed for EC computational pathology. Unlike earlier CNN-based approaches capturing local image features, EndoNet’s vision-transformer architecture extracts local and global histological patterns from weakly annotated whole-slide images. Its attention-based design enhances interpretability by highlighting relevant tissue regions and reducing extensive pixel-level annotation needs. The model showed strong EC grading performance and generalizability across external TCGA cohorts, particularly for endometrioid grade 1, grade 3, and serous carcinoma. However, challenges remain for intermediate-grade tumours and integration with non-morphological clinical or molecular data (Goyal et al. 2024).

**EndoRisk** is a preoperative risk stratification framework designed to predict lymph node metastasis (LNM) and disease-specific survival in EC using a Bayesian network (Schoenaker et al. 2025). The original ENDORISK-I model was developed using a multicentre cohort of 763 patients and externally validated in two prospective cohorts. Unlike conventional regression-based models, the Bayesian network structure can capture probabilistic dependencies among clinical, histopathological, molecular, laboratory, imaging, and cytology variables, while retaining functionality when some information is incomplete. ENDORISK-I included variables such as tumour grade, ER/PR status, p53 expression, L1CAM expression, CA125 serum level, thrombocyte count, imaging findings, and cervical cytology. The model achieved good predictive performance in validation cohorts, although performance was lower in aggressive subtypes (Reijnen et al. 2020). ENDORISK-II further refined this framework by incorporating molecular classification and preoperative myometrial invasion assessment, improving personalized risk prediction and clinical interpretability (Vinklerová et al. 2022).

**HECTOR** (Histopathology-based Endometrial Cancer Tailored Outcome Risk) represents a multimodal deep learning approach for recurrence-risk prediction using H&E-stained whole-slide images. The model integrates histopathological image features, image-derived molecular classification, and anatomical stage to improve prognostic stratification beyond conventional clinicopathological assessment. Trained on data from 2,072 patients across multiple randomized trials, HECTOR showed strong prognostic performance and clear separation of recurrence-risk groups. It also provided explainable outputs linked to morphological features and suggested potential utility in predicting benefit from adjuvant chemotherapy. However, before broad clinical deployment, such models require validation across diverse real-world settings, including variation in tissue processing, staining protocols, scanner hardware, and staging practices across institutions (Volinsky-Fremond et al. 2024).

## Multi-modality: bridging histology, imaging, and genomics

**Fig. 5:**
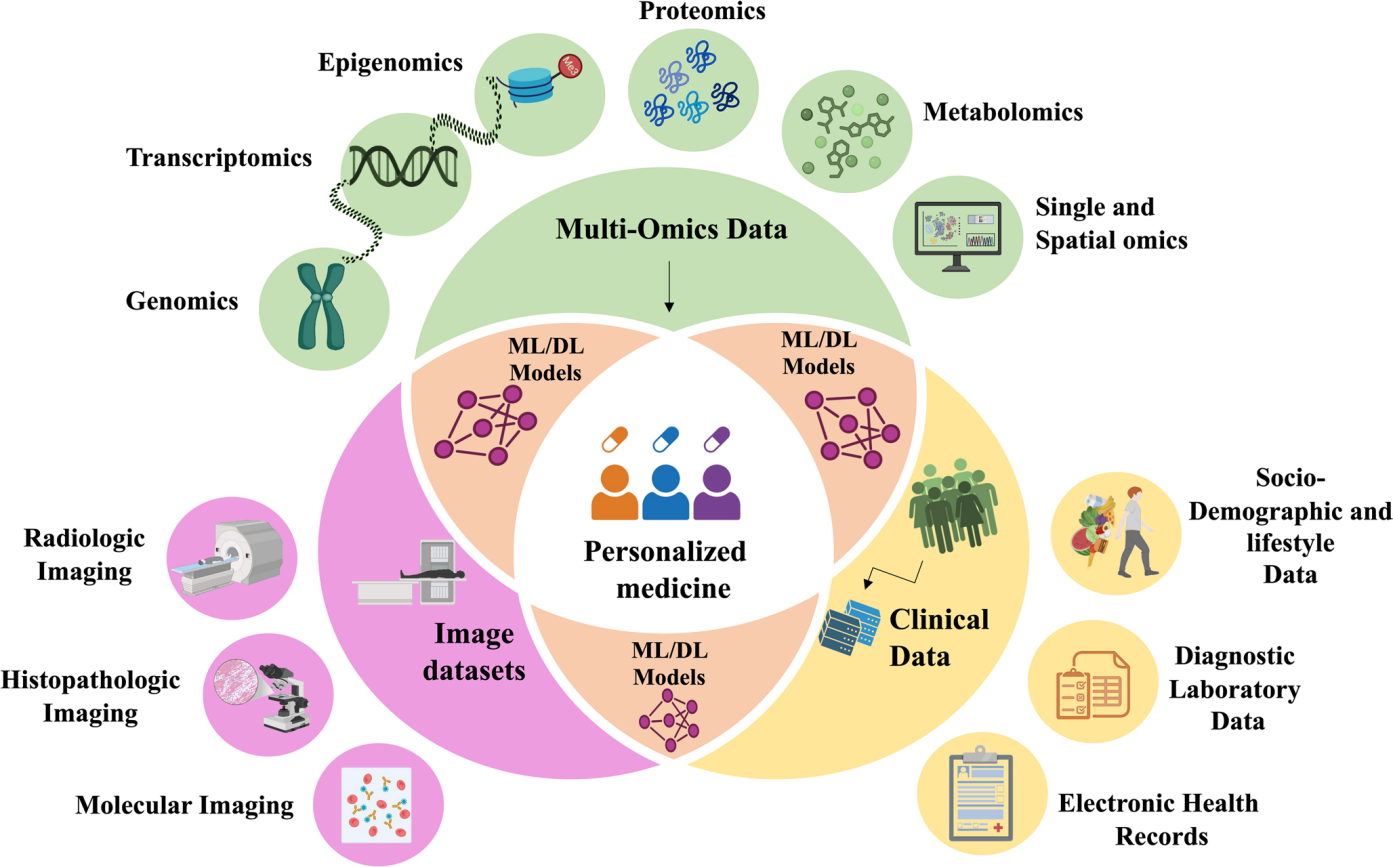
Overview of multimodal data fusion for AI-enabled precision oncology in Endometrial Cancer. Integration of molecular profiling, imaging data, and clinical information through artificial intelligence is expected to enable improved diagnosis, risk stratification, and treatment selection, supporting personalized management of endometrial cancer.

In biomedical research, data modality refers to patient-derived information, such as clinical data, imaging, histopathology, or molecular profiles, and two or more modalities constitute a multimodal dataset (Baltrusaitis et al. 2019; Sleeman et al. 2022) (Fig. 5). Traditionally, EC research and clinical decision-making have relied on single-modality analyses, which provide partial insights but fail to capture the full biological and clinical complexity of the disease. Tumour morphology, molecular alterations, imaging features, and clinical behaviour often show incomplete concordance, limiting accurate risk stratification when assessed in isolation (Waqas et al. 2024). Integrating histology, imaging, genomics, and clinical data enables a more comprehensive understanding of ec biology and improves clinical correlation. in practice, imaging modalities such as ultrasound, CT, MRI, and PET/CT inform tumour extent, invasion, and metastasis and guide treatment planning (Daoud et al. 2025). AI-driven multimodal models further enhance prognostic and risk assessment by combining imaging with histopathological, molecular, and clinical data, leading to more precise and individualized decision-making (Table 4). For example, a multimodal AI system incorporated intermediate integration of histopathology WSIs features (extracted from CNN) and clinical data (encoded via MLP) to enhance sensitivity and specificity. the performance of this multimodal approach was superior to unimodal approaches, securing an accuracy score of 0.91 and an auc of 0.95 (Dass mazumdar et al. 2026). These findings demonstrate the value of multimodal fusion for early-stage ec diagnosis.

**Table 4:** Case studies incorporating multi-modal data and deep learning in endometrial cancer prognosis

Study	Modalities	Cohort	Fusion strategy	AI application	Results/conclusions	Reference
CNNs-based	Ultrasound + Doppler + clinical data	1,443 images from 611 patients (132 EC, 479 non-EC)	*Intermediate fusion* (Feature extraction → Integration→ Classification)	DL (ResNet, EfficientNet, DenseNet) + clinical data	External AUC 0.892 improved EC detection	(Lin et al. 2025)
Swin Transformer	Multi-view MRI T2WI	122 early EC patients 1865 T2WI	*Intermediate (feature level) fusion* 3 T2WI MRI views →feature extraction → feature fusion → IA/IB staging	Transformer-based MRI fusion staging	IA vs. IB staging, accuracy 1.0	(Zheng et al. 2025)
DL-based subtyping	DL + histopathology + multi-omics	138 EC + 20 normals; 10 omics platforms	*Intermediate fusion* Multi-omics + clinical data → feature fusion → biomarker discovery	DL histopathology analysis	Identified PIK3R1, CTNNB1 markers	(Dou et al. 2023)
TF-DWGNet	mRNA + miRNA expression	TCGA BRCA, UCEC, and KIPAN	*Intermediate fusion* Multi-omics → Graph construction → GNN fusion → subtyping	GEDFN (Graph-Embedded Deep Feedforward Network) + Tensor Fusion	Accuracy 0.821 → BRCA (highest)	(Yang and Chen 2025)

## Current applications of multi-omics: from diagnosis to therapy

### Early detection, liquid biopsies, and clinical transition

Early detection is a major determinant of clinical outcomes in EC. Conventional diagnostics, such as tissue and surgical biopsies, are invasive, poorly suited for longitudinal monitoring, and provide only a static, spatially limited view of tumour biology, often failing to capture intra-tumour heterogeneity and molecular evolution (Yu et al. 2021). Liquid biopsy has emerged as a transformative alternative, enabling minimally invasive, real-time monitoring of tumour dynamics and treatment response through repeated sampling (Lone et al. 2022).

Beyond blood-based assays, regionally derived non-blood liquid biopsies, particularly cervicovaginal fluid (CVF) and urine, offer superior sensitivity in early-stage disease by overcoming low circulating tumour signal burdens (O’Flynn et al. 2021). Proteomic profiling of CVF using SWATH-MS combined with ML achieved robust discrimination between EC patients and symptomatic controls (AUC 0.92; accuracy 86%), highlighting its potential as a non-invasive screening tool (Njoku et al. 2024). The accessibility of samples obtained via tampons(Bakkum-Gamez et al. 2015) or vaginal swabs further enhances the feasibility and cost-effectiveness (Wen et al. 2022).

In blood-based diagnostics, AI and ML have markedly improved analytical performance. Ensemble ML applied to metabolomic profiles demonstrated > 99% accuracy in EC screening among postmenopausal women (Troisi et al. 2020). Circulating tumor DNA (ctDNA) has also proven useful for detecting minimal residual disease and monitoring recurrence (Recio et al. 2024), while AI-assisted analysis of circulating cell-free DNA (cfDNA) (Thalambedu et al. 2025) may enable earlier detection of malignancy and disease progression (Tsui et al. 2025).

Commercialized assays are increasingly shaping the clinical management of EC. Foundation One CDx (F1CDx), an FDA-approved comprehensive genomic profiling (CGP) tissue assay, is widely used to guide targeted therapy and immunotherapy decisions in advanced EC through assessment of tumour mutational burden (TMB) and microsatellite instability (MSI) (Pinet et al. 2023). In parallel, the FoundationOne Liquid CDx platform enables liquid-based genomic profiling by analysing circulating cell-free DNA (cfDNA) from peripheral blood, thereby identifying actionable mutations and resistance-associated variants without requiring an active tissue biopsy (Woodhouse et al. 2020). Expanding beyond disease management into early detection, multi-cancer early detection (MCED) strategies have also gained prominence (Imai et al. 2025). The landmark GRAIL Circulating Cell-free Genome Atlas (CCGA) trial (NCT02889978) investigated a cfDNA methylation-based blood assay across more than 50 cancer types, including EC (Klein et al. 2021). Although the Galleri assay detects generalized cancer signals, its sensitivity for early-stage (Stage I/II) EC remains limited because of low tumour DNA shedding into circulation. This limitation has driven the development of uterine-focused multi-omic liquid biopsy approaches (Di Carlo 2026). Ongoing studies, including the LUSTRE trial, are evaluating uterine-proximal assays combining cfDNA mutation profiling with protein biomarkers for rapid triaging of postmenopausal bleeding (Feng et al. 2021). In parallel, personalized Signatera ctDNA assays are being investigated for early detection of molecular recurrence prior to conventional CT or MRI imaging (Magbanua et al. 2021).

### Molecular subtype-stratified prediction of therapy response

Molecular subclassification in EC has progressed from prognostic stratification to a predictive framework guiding therapeutic decisions in both adjuvant and recurrent settings. EC harbours the highest prevalence of dMMR among solid tumours, conferring marked susceptibility to immune checkpoint inhibition (Le et al. 2017). Contemporary trials increasingly adopt molecularly stratified designs. The phase III NRG-GY018 trial demonstrated significant progression-free survival (PFS) benefit with first-line pembrolizumab plus chemotherapy in advanced or recurrent dMMR EC (Eskander et al. 2023). Similarly, the RAINBO/p53abn-RED trial evaluates the addition of olaparib to chemotherapy in p53-abnormal EC. Therapeutic de-escalation has also been explored in favourable molecular subgroups. The *POLE*mut-BLUE phase II trial assessed the omission of adjuvant therapy in *POLE*-mutated EC, reflecting the exceptionally favourable prognosis of this subtype (Bosse et al. 2023).

Parallel advances in computational pathology and radiomics have enabled non-invasive prediction of molecular features from routine histopathological and imaging data. Deep learning analysis of H&E whole-slide images using multi-resolution architectures, such as EfficientNets, predicted MMR deficiency with an AUC of 0.821, supporting the potential of digital pathology for immunotherapy stratification (Whangbo et al. 2024). Similarly, CE-CT-based radio genomics combining intra- and peritumoural features predicted MMR-D and TMB-high ECs with AUROC values of 0.78 and 0.87, respectively (Veeraraghavan et al. 2020). Complementing these approaches, a multiparametric MRI-radiomics model predicted MSI status with an external validation AUC of 0.862, highlighting the potential of AI-driven imaging for non-invasive molecular stratification in EC (Jia et al. 2024).

### Drug discovery and target identification

Beyond clinical trials, ML-based predictive frameworks integrate gene expression profiles with drug sensitivity data from large pharmacogenomic repositories such as Genomics of Drug Sensitivity in Cancer (GDSC) (Wang et al. 2025a, b, c, d) and the Library of Integrated Network-Based Cellular Signatures (LINCS) (Zhang et al. 2022). Integration of transcriptomic(Wang et al. 2024c) and proteomic datasets has enhanced drug response prediction and biomarker discovery (Yasar et al. 2024). Recently, novel multi-omics ML frameworks combining mRNA, miRNA, and methylation data have significantly improved EC molecular stratification as well as the identification of potential therapeutic biomarkers (*CDKN2A*,* MLH1*,* PP4R4*, and has-miR-378a) (Patel et al. 2026). Advances in DL, particularly integration of deep reinforcement learning (DRL), generative adversarial networks (GANs), and variational autoencoders (VAEs) with molecular docking and dynamics studies, have enabled the design and screening of novel peptide-based inhibitors targeting key EC-associated proteins (AKT1, ESR1, CTNNB1, and Connexin-43) (Fatima et al. 2026). In addition, heterogeneous graph-based DL models exhibit high levels of predictability in drug-target interaction prediction with AUC and AUPR values of 0.870 and 0.872, respectively (Li et al. 2024a). DL approaches can now infer microsatellite instability (MSI) status and tumour mutation burden directly from histopathological images and multi-omics data, enabling rapid, non-invasive identification of patients most likely to benefit from immunotherapy (Wang et al. 2024b; Liu et al. 2025b). Immunological biomarkers identified using ML/DL approaches, including their data sources, modelling frameworks, and clinical relevance, are summarised in Table 5.

**Table 5:** Compilation of Immunological Biomarkers identified using ML/DL approaches

Immunological biomarkers	Biological significance	Data source	ML/DL model	Objective of the study	Key findings	Reference
TMB (high/low)	Predicts ICI response	H&E WSI	TR-MAMIL framework	TMB & subtype prediction	73% MeanSS; AUROC 82% → patients with better DSS/OS	(Wang et al. 2024a)
MMRd, *POLE*mut, p53abn	Molecular classes for risk & treatment	929 internal + 100 TCGA WSIs	EndoNet: a hybrid model using CNNs (ResNet-18)	Grade classification & MSI prediction	High concordance with molecular testing; reliable MSI prediction	(Goyal et al. 2024)
*FLT1* (Fms-like tyrosine kinase 1)	Angiogenesis marker linked to OS	CPTAC + TCGA	XGboost	Radiomics-based OS prediction	AUC (up to 0.885); FLT1 is highly correlated with the model output	(Zhang et al. 2025b)
NK cells, CD4 + T cells, monocytes, and IFN-γ	Immune infiltration linked to recurrence	TCGA-UCEC	Random Forests	Recurrence prediction	Accuracy 68.6%; identifies relapse-prone low-risk patients	(Bruno et al. 2023)
*LTB*,* GPR18*,* BATF*,* ACAP1*,* GRAP2*,* and CTSW*	Immune phenotype & poor response	TCGA UCEC	LASSO + RSF	Prognosis & immunotherapy response	High IRRS → poor OS; reduced anti-PD1 response	(Wang et al. 2024c)
*TMEM150B*,* CACNA2D2*,* TRPM5*,* NOL4*,* CTSW*,* and SIGLEC1*	T-cell activation & immune infiltration	TCGA UCEC + TCGA-CDR	LASSO RSF	Immune-related prognostic genes	High immune/stromal scores → better OS/PFI	(Chen et al. 2020)

## Practical challenges in building clinically deployable AI models

### Data heterogeneity and external validation

A major barrier to clinical translation of ML models in EC is data heterogeneity arising from variability in tissue fixation, biopsy vs. hysterectomy specimen handling, sequencing platforms, MRI/CT imaging protocols across vendors, clinical annotation, and diverse patient demographics (Liu and Cui 2025). Many EC datasets also lack long-term oncological follow-up or disproportionately represent specific molecular subtypes, limiting their utility for recurrence prediction, treatment-response modelling, and clinical decision support. Therefore, clinically deployable EC models require standardized pipelines for staining, scanning, imaging acquisition, molecular profiling, and annotation harmonization, together with external validation in independent cohorts, consistent with transparent reporting principles for clinical prediction models (Collins et al. 2015). EC-specific solutions should address both technical heterogeneity and disease-specific imbalance. Public and institutional datasets often overrepresent common endometrioid and NSMP tumours while underrepresenting *POLE*-mutated tumours, p53-abnormal tumours, aggressive non-endometrioid histology, and minority populations (Kumari et al. 2025). Model development should therefore include subtype-stratified sampling, batch-effect correction for omics data, harmonized pathology/imaging preprocessing, and subgroup-level performance reporting for *POLE*mut, MMRd, NSMP, and p53abn tumours. In parallel, XAI methods such as SHAP, LIME, attention maps, and saliency-based visualisation should verify that predictions are driven by clinically meaningful features, including myometrial invasion, tumour architecture, immune infiltration, MMR/p53-related morphology, and molecular subtype-associated patterns, rather than technical artefacts (Ekanayake et al. 2022; Salih et al. 2025).

### EC-specific class imbalance and emerging solutions

Class imbalance is highly pronounced in EC datasets, particularly when contrasting benign endometrial hyperplasia with malignant tissue, or when isolating rare, aggressive phenotypes like serous or clear cell carcinomas. For example, TCGA endometrial transcriptomic data includes > 500 tumour samples but only ~ 35 normal samples, predisposing models to bias towards the majority class (Getz et al. 2013, 2025). To mitigate this, data-level strategies such as oversampling (e.g., SMOTE, ADASYN, Borderline-SMOTE)(Viloria et al. 2020; Mohammed et al. 2020; Elreedy et al. 2023) and undersampling (e.g., Tomek links, cluster centroids) are widely employed, although they risk overfitting or information loss (Devi et al. 2017; Lin et al. 2017; Kaur and Gosain 2018).

Generative approaches, including generative adversarial networks (GANs) and hybrid models such as E-GAN, offer more realistic minority-class synthesis but remain under clinical evaluation (Suresh et al. 2023; Su et al. 2024). Advanced adaptive oversampling methods (e.g., SASMOTE, RSMOTE, CDSMOTE) (Pradipta et al. 2021; Elyan et al. 2021; Kosolwattana et al. 2023) and hybrid ensemble strategies (e.g., SMOTEBoost, RUSBoost) further enhance minority-class representation (Chawla et al. 2003; Seiffert et al. 2010; Han et al. 2020). Algorithm-level solutions, including cost-sensitive learning tailored to the penalty of missing high-grade EC malignancies, alongside stratified deep neural networks (Sadaiyandi et al. 2023), and automated meta-learning frameworks like ATOMIC, enable joint optimization of resampling and classifier selection to preserve accuracy across all EC sub-cohorts (Moniz and Cerqueira 2021).

### Clinical accountability and explainable AI (XAI)

While DL architectures have improved predictive accuracy, they often suffer from limited interpretability. This “black box” nature is particularly problematic in clinical settings, where transparency and trust are prerequisites for adoption and regulatory approval (Gulum et al. 2021). Explainable AI (XAI) techniques, including SHapley Additive exPlanations (SHAP) and Local Interpretable Model Agnostic Explanations (LIME), address this limitation by quantifying feature contributions and elucidating decision pathways, thereby enhancing model transparency, accountability, and clinician trust (Salih et al. 2025). This transforms AI outputs into interpretable, auditable data points, fostering the transparency and trust required for regulatory approval and safe integration into gynaecologic oncology decision-support systems.

## Future directions: AI-enabled precision management of EC

### Transformer-based models for histopathology and molecular subtyping

EC histopathology often shows spatial and morphological heterogeneity, which can limit CNN-based models that mainly capture local image features. Transformer-based models and multiple-instance learning (MIL) frameworks can model long-range contextual relationships across whole-slide images, making them useful for WSI-based molecular subtype prediction and tumour mutation burden estimation. Transformer-based multiple instance learning (MIL) models overcome this constraint by capturing long-range contextual dependencies within WSIs (Goyal et al. 2023). The ETMIL-SSLViT framework, integrating self-supervised Vision transformer (ViT) features, accurately inferred EC molecular subtypes and tumour mutation burden directly from H&E slides. Evaluated on 918 EC and 1,495 colorectal cancer slides from TCGA, this approach significantly outperformed seven state-of-the-art CNN models, demonstrating strong concordance with genomic ground truth (*p* < 0.001) (Wang et al. 2025a, b, c, d). Hybrid ResNet50-ViT architectures have also shown promise in radiological imaging. A ResNet50–ViT model achieved superior classification of benign, malignant, and normal endometrial lesions, with accuracies of 90.24% on MRI and 86.99% on CT, highlighting the particular advantage of transformer-based models for MRI-driven EC detection (Abu-azzam et al. 2025).

### Self-supervised learning for data-efficient modelling

The scarcity of large, well-annotated EC datasets has accelerated the adoption of self-supervised learning (SSL), which enables robust representation learning without extensive manual annotation (Liu et al. 2025a). A self-supervised triplet contrastive learning (SSTCL) framework incorporating mosaic masking and bottleneck transformers achieved 77–83% accuracy using only 20–100% labelled data on public datasets and 96.8% accuracy on institutional cohorts (Zhao et al. 2023). In a complementary pan-cancer study, SSL combined with attention-based MIL enabled mutation prediction from H&E slides across TCGA and CPTAC cohorts. TCGA-UCEC notably exhibited the highest predictability, with key driver mutations (including *PTEN* and *TP53*) achieving AUROC values near 0.70, suggesting SSL can unlock distinct, highly learnable morpho-genomic signatures inherent to EC tissue architecture without requiring high-cost sequencing (Saldanha et al. 2023).

### Graph attention networks for tumour microenvironment modelling

Endometrial tumours are complex ecosystems where tumour cells constantly interact with surrounding immune cells, stromal components, and the extracellular matrix. Graph attention networks (GATs) facilitate explicit modelling of these complex cell–cell interactions, TME architecture, and multi-omics dependencies. Frameworks such as scBGDL integrate single-cell and bulk transcriptomic data, achieving superior prognostic performance across TCGA cohorts (Liu et al. 2025c). Beyond prognosis, graph-based DL has been applied to drug discovery; the DTRE model, integrating graph convolutional networks, GATs, and attention mechanisms, demonstrated strong drug–target interaction prediction (AUC = 0.87), addressing a major bottleneck in therapeutic development tailored to specific EC profiles (Li et al. 2024a). Similarly, omicsGAT leverages multi-head attention to weight neighbourhood relevance in RNA-seq graphs, improving phenotype prediction, patient stratification, and cellular clustering across large-scale gynaecologic oncology datasets (Baul et al. 2022).

### Digital twins for adaptive treatment simulation

Digital Twins (DTs), dynamic virtual representations of patients continuously updated with real-world data, are emerging as a paradigm for precision oncology. By integrating multimodal and multiscale data streams (clinical, genomic, and histopathological) and cancer centres, DTs enable adaptive prognostic and therapeutic simulations that reflect a patient’s evolving disease state (Mollica et al. 2024). In EC management, early efforts are underway to develop DT frameworks in collaboration with tertiary cancer centres, aiming to support personalized risk stratification and treatment optimization (Kaul et al. 2023).

### Federated learning for multi-center gynecologic oncology consortia

Since single-institution EC data collections are rarely large enough to train unbiased models, and data-sharing is restricted by strict data-privacy regulations, Federated learning (FL) offers a decentralized path forward (Daram 2025). Specifically, local models are trained at each site, with only model parameters exchanged and aggregated using algorithms such as FedAvg, aligning FL with regulatory frameworks including the Health Insurance Portability and Accountability Act (HIPAA) and the General Data Protection Regulation (GDPR) (McMahan et al. 2017). The utility of this approach in EC was demonstrated by the FedCMC model, which applied FL to predict deep myometrial invasion across three centers (*n* = 902), achieving AUCs of 0.83–0.90 and outperforming prior federated approaches by improving classification accuracy by 10.6% and fairness by 31.2% (Li et al. 2025a). Moving forward, widespread deployment of FL in EC research will require efficient communication protocols, harmonized preprocessing across scanners and platforms, and continuous monitoring to detect local model drift over time.

## Ethical and regulatory considerations

The clinical translation of AI and multi-omics analytics in EC raises substantial ethical and regulatory challenges. Data privacy and security are critical, as models rely on sensitive histopathological, imaging, and genomic data, creating risks of re-identification and leakage, even under privacy-preserving approaches such as federated learning, which still require safeguards like secure aggregation and differential privacy (Bussola et al. 2021; Tabakhi et al. 2022). Algorithmic bias is another major concern, as models may reflect demographic, clinical, or molecular imbalances in training data, leading to unequal performance and potential amplification of health disparities; this necessitates diverse, representative datasets and systematic bias auditing (Char et al. 2018). Beyond these data-related challenges, real-world adoption of multi-omics AI models is also limited by infrastructure and modality availability. Many routine clinical settings may not have standardized access to mass-spectrometry proteomics, spatial transcriptomics, digital pathology scanners, harmonized MRI protocols, or personnel trained to manage integrated molecular–imaging data pipelines. These constraints partly explain why models with strong retrospective performance are not yet widely implemented in routine EC care. To improve clinical feasibility, future workflows should prioritise routinely available inputs, such as H&E-stained slides, pelvic MRI, CT, and standard clinicopathological variables, while using cross-modal learning approaches to infer or approximate high-cost molecular features where appropriate (Panayides et al. 2020).

Despite promising reported performance in some studies, including AUC values approaching 0.90, a clear implementation gap remains between retrospective model development and routine clinical deployment. Limited interpretability, especially in complex multimodal DL architectures, remains a barrier to clinician trust, ethical accountability, and regulatory approval. Therefore, explainable AI (XAI), prospective validation, standardized reporting, workflow integration, and post-deployment monitoring should be considered essential requirements for translating AI-enabled multi-omics models into clinical EC management. While medical imaging represents the most mature regulatory pathway for AI, emerging applications such as Digital twins introduce unresolved issues around informed consent, data ownership, and patient autonomy (Pesapane et al. 2018). Finally, robust clinical validation, transparent reporting, external testing across populations, and continuous post-deployment monitoring are essential to ensure real-world safety, reliability, and sustained patient protection.

## Review limitations

Several limitations of this review article should be acknowledged. First, this article is a narrative synthesis rather than a systematic review or meta-analysis and, therefore, may be subject to selection bias. Second, because of the broad scope of AI-enabled multi-omics in EC, we prioritised studies with direct relevance to AI, multi-omics integration, external validation, and clinical translation, while some early-stage method-development studies and isolated single-omics analyses may not have been discussed in detail. Third, the focus on peer-reviewed English-language literature may have excluded relevant non-English studies or recent preprints. Fourth, although this review discusses single-cell RNA sequencing and spatial transcriptomics, other emerging technologies, including spatial proteomics, single-cell methylomics, miRNA-seq, cell-free RNA profiling, and AI-assisted pathology quality-control systems, were not covered extensively. Finally, the field still lacks standardized reporting, prospective validation, and harmonized benchmarking across datasets, which limits direct comparison of model performance across studies. These limitations highlight the need for regular updates as AI-enabled multi-omics approaches continue to mature in EC precision oncology.

## Conclusion

EC is a biologically heterogeneous disease in which conventional histopathology, imaging, and single-layer molecular approaches may not fully capture the complexity needed for individualized risk assessment and treatment selection. Molecular classification has improved EC stratification, but its clinical impact can be strengthened by integrating genomics, transcriptomics, proteomics, metabolomics, imaging, histopathology, and clinical data through AI-enabled multi-omics approaches. ML and DL models are increasingly used for biomarker discovery, molecular subtyping, recurrence prediction, treatment-response modelling, and non-invasive inference of clinically relevant molecular features.

Despite these advances, translation into routine EC care remains limited by dataset heterogeneity, small and imbalanced cohorts, incomplete external validation, batch effects, limited interpretability, and regulatory and infrastructure barriers. Future progress requires standardized data harmonization, transparent reporting, prospective multicentre validation, explainable AI, and clinically feasible workflows relying on routinely available data where possible. With these safeguards, AI-enabled multi-omics may help move EC research beyond biomarker discovery toward clinically actionable precision oncology.

## Supplementary Information

Below is the link to the electronic supplementary material.

Supplementary Material 1 (DOCX 180 KB)

## Data Availability

No datasets were generated or analysed during the current study.

## Abbreviations

EC: Endometrial Cancer
AI: Artificial Intelligence
ML: Machine Learning
DL: Deep Learning
TCGA: The Cancer Genome Atlas
CPTAC: Clinical Proteomic Tumour Analysis Consortium
GEO: Gene Expression Omnibus
GTEx: Genotype-Tissue Expression Project
HPA: Human Protein Atlas
SRA: Sequence Read Archive
FIGO: International Federation of Gynecology and Obstetrics
ESGO: European Society of Gynaecological Oncology
ESTRO: European Society for Radiotherapy and Oncology
ESP: European Society of Pathology
ProMisE: Proactive Molecular Risk Classifier for Endometrial Cancer
MMR: Mismatch Repair
MMRd: Mismatch Repair Deficient
dMMR: Deficient Mismatch Repair
MSI: Microsatellite Instability
NSMP: No Specific Molecular Profile
IHC: Immunohistochemistry
TME: Tumour Microenvironment
NGS: Next-Generation Sequencing
scRNA-seq: Single-Cell RNA Sequencing
ST: Spatial Transcriptomics
PD-1: Programmed Cell Death Protein 1
TCGA-UCEC: TCGA – Uterine Corpus Endometrial Carcinoma
EMT: Epithelial–Mesenchymal Transition
DDR: DNA Damage Response
PI3K: Phosphoinositide 3-Kinase
AKT: Protein Kinase B
mTOR: Mechanistic Target of Rapamycin
MAPK: Mitogen-Activated Protein Kinase
TCA: Tricarboxylic Acid Cycle
RF: Random Forest
LASSO: Least Absolute Shrinkage and Selection Operator
Cox: Cox Proportional Hazards Model
SVM: Support Vector Machine
KNN: K-Nearest Neighbour
PCA: Principal Component Analysis
t-SNE: t-Distributed Stochastic Neighbour Embedding
UMAP: Uniform Manifold Approximation and Projection
NMF: Non-negative Matrix Factorization
RFE: Recursive Feature Elimination
AUC: Area Under the Curve
SMOTE: Synthetic Minority Oversampling Technique
ADASYN: Adaptive Synthetic Sampling
SHAP: SHapley Additive exPlanations
LIME: Local Interpretable Model-agnostic Explanations
LR: Logistic Regression
RSF: Random Survival Forest
TMB: Tumour Mutational Burden
SWATH-MS: Sequential Window Acquisition of All Theoretical Mass Spectra
UHPLC-HRMS: Ultra-High-Performance Liquid Chromatography–High-Resolution Mass Spectrometry
TIDE: Tumour Immune Dysfunction and Exclusion
EaSleR: Ensemble Approach for Immune Response Prediction
TIMER: Tumour Immune Estimation Resource
CD8: Cluster of Differentiation 8
CD4: Cluster of Differentiation 4
IFN-γ: Interferon Gamma
NK: Natural Killer
OS: Overall Survival
DSS: Disease-Specific Survival
PFI: Progression-Free Interval
PFS: Progression-Free Survival
LNM: Lymph Node Metastasis
CVF: Cervicovaginal Fluid
ctDNA: Circulating Tumour DNA
cfRNA: Cell-Free RNA
cfDNA: Circulating cell-free DNA
GDSC: Genomics of Drug Sensitivity in Cancer
LINCS: Library of Integrated Network-Based Cellular Signatures
MOFA: Multi-Omics Factor Analysis
SNF: Similarity Network Fusion
CCA: Canonical Correlation Analysis
sCCA: Sparse Canonical Correlation Analysis
DIABLO: Data Integration Analysis for Biomarker Discovery Using Latent Variable Approaches for Omics Studies
MOLI: Multi-Omics Late Integration
HNMDRP: Heterogeneous Network-based Model for Drug Response Prediction
MLP: Multilayer Perceptron
CNN: Convolutional Neural Network
RNN: Recurrent Neural Network
GNN: Graph Neural Network
GAT: Graph Attention Network
GCN: Graph Convolutional Network
LSTM: Long Short-Term Memory
GRU: Gated Recurrent Unit
WSI: Whole Slide Image
MRI: Magnetic Resonance Imaging
CT: Computed Tomography
PET: Positron Emission Tomography
ECC: Endometrial Cell Clumps
MIL: Multiple Instance Learning
ViT: Vision Transformer
SSL: Self-Supervised Learning
SSTCL: Self-Supervised Triplet Contrastive Learning
GAN: Generative Adversarial Network
E-GAN: Evolutionary Generative Adversarial Network
SASMOTE: Self-Adaptive Synthetic Minority Oversampling Technique
RSMOTE: Random Synthetic Minority Oversampling Technique
CDSMOTE: Cluster-Based Dynamic SMOTE
RUSBoost: Random Under Sampling Boost
SMOTEBoost: Synthetic Minority Oversampling Technique Boost
ATOMIC: Automated Meta-Learning Framework for Class Imbalance
XAI: Explainable Artificial Intelligence
FL: Federated Learning
DT: Digital Twins
FedAvg: Federated Averaging Algorithm
HIPAA: Health Insurance Portability and Accountability Act
GDPR: General Data Protection Regulation
