# Supplementary material for: Integrating artificial intelligence and multi-omics data for precision oncology in endometrial cancer: a narrative review
Source: Funct Integr Genomics. 2026 Jun 29;26(1):168. doi: 10.1007/s10142-026-01957-2 (PMC13310831; doi:10.1007/s10142-026-01957-2)
Supplement: Supplementary file 1 — Supplementary Material 1 (DOCX 180 KB) [file 10142_2026_1957_MOESM1_ESM.docx]

**Integrating Artificial Intelligence and Multi-Omics Data for Precision Oncology in Endometrial Cancer: A Narrative Review**

Oishee Mondal^1¥^, Masuma Khatun^¥2,3^, Ankita Lawarde^3^, Sajitha Lulu S^1^, [Vino Sundararajan](https://link.springer.com/article/10.1186/s13072-025-00595-5#auth-Vino-Sundararajan-Aff1) ^1^, Andres Salumets ^3,4,5^*, Vijayachitra Modhukur^3,4^*

1. Integrative Multiomics Lab, School of Bio Sciences and Technology, Vellore Institute of Technology, Vellore – 632014, Tamil Nadu, India
2. Department of Obstetrics and Gynecology, Helsinki University Hospital and University of Helsinki, Haartmaninkatu 8, 00290, Helsinki, Finland
3. Department of Obstetrics and Gynecology, Institute of Clinical Medicine, University of Tartu, Tartu, Estonia
4. Celvia CC AS, Tartu, Estonia
5. Division of Obstetrics and Gynaecology, Department of Clinical Science, Intervention and Technology (CLINTEC), Karolinska Institutet, and Karolinska University Hospital, Stockholm, Sweden

^¥^contributed equally to this work.

*Corresponding author(s):

Dr. Vijayachitra Modhukur, Department of Obstetrics and Gynecology, Institute of Clinical Medicine, University of Tartu, L. Puusepa 8, 50406 Tartu, Estonia. E-mail: [modhukur@ut.ee](mailto:modhukur@ut.ee);

Prof. Andres Salumets, Division of Obstetrics and Gynaecology, Department of Clinical Science, Intervention and Technology (CLINTEC), Karolinska Institutet, and Karolinska University Hospital, Stockholm, Sweden. Email: [andres.salumets@ki.se](mailto:andres.salumets@ki.se)

**Supplementary Tables**

**Table S1: Key Multi-omics Endometrial Cancer Studies Using Public Databases and Computational Approaches**

| **Publicly Available Databases** | **Computational tools/Algorithms used** | **Integration type** | **Validation Layer** | **Outcome** | **Reference** |
| --- | --- | --- | --- | --- | --- |
| TCGA, GEO,  CPTAC | Consensus clustering;  LASSO; Random Forest (RF); Cox | Sequential integration (transcriptomics-driven subtype discovery) | External transcriptomics cohort (GEO) + proteomic (CPTAC), cell-line validation (CCLE) | 13-gene prognostic model | (Liu et al. 2024) |
| TCGA; GEO (scRNA-seq) | Ensemble survival ML | Sequential multi-modal  (Bulk RNA → scRNA → prognostic model) | scRNA + in-vitro validation | 7-gene pyroptosis prognostic signature | (Huang et al. 2025a) |
| TCGA, GTEx, GEO, HPA | Statistical and enrichment analysis | Targeted multi-omics characterization | External Transcriptomics cohort (GEO) + qRT-PCR | Prognosis (KLRG2) and immune suppression | (Huang et al. 2025b) |
| TCGA, GEO, SRA | Regression -based ML | Feature-level integration | External CPTAC cohort (N=217 UCEC tumors) | ARID1A-based stratification | (Song et al. 2025) |
| TCGA, GEO, CPTAC, GTEx | Cox and survival analysis | Gene-centric transcriptomics-proteomics characterisation | External transcriptomics cohort (GEO) + CPTAC validation | S100A2 → poor prognosis | (Zhang et al. 2022b) |
| TCGA, GEO, SRA | TIDE; EaSleR; ImmunoPhenoScore | Late multi-omics integration | Transcriptomics (GEO) + scRNA + independent immunotherapy cohort (n=12) + IHC + Flow cytometry + macrophage co-culture functional assays | Immune active vs suppressed TME predicts responders | (Guan et al. 2025) |
| TCGA, GTEx, CPTAC, NCBI | Statistical & immune profiling | Single-gene multi-omics analysis | Cross-database validation (TCGA, GTEx, HPA, CPTAC, cBioPortal, TIMER2.0) + No clinical cohort /wetlab | S1PR1↓ in EC (no prognostic impact) | (Xiong et al. 2025) |

| **Metric** | **Formula** | **Interpretation** | **Key Features** | **Reference** |
| --- | --- | --- | --- | --- |
| **Accuracy** | $\frac{TP+TN}{TP+TN+FP+FN}$ | Proportion of correctly classified samples among all instances | Measure overall correctness, but not ideal for imbalanced data | (Chen et al. 2020) |
| **Recall** | $\frac{TP}{TP+FN}$ | Sensitivity or True positive rate → measures actual positives correctly identified | Important for detecting minority or positive cases; high recall → few false negatives | (Vela Moreno et al. 2025) |
| **F1-Score** | $\frac{2*Precision*Recall}{Precision+Recall}$ | The mean of precision and recall balances the trade-off between them | Suitable for imbalanced data; balances false positives and false negatives | (Goyal et al. 2024) |
| **Balanced accuracy** | $\frac{Recall+Specificity}{2}$ | Average of sensitivity (recall) and specificity (true negative rate) | Adjusted for imbalance; can reflect positive + negative class performance | (Bruno et al. 2023) |
| **Precision** | $\frac{TP}{TP+FP}$ | Proportion of predicted positives that are truly positive | High precision indicates few false positives | (Miller et al. 2024) |
| **Area Under Curve (AUROC)** | — | Plots True Positive Rate vs. False Positive Rate across thresholds. Ranges from 0 (poor) to 1 (excellent). | Indicates ranking ability; higher AUROC means better separability of classes | (Miller et al. 2024) |
| **Area Under Precision-Recall Curve** | — | Precision vs recall across thresholds; higher values indicate strong performance on the positive (minority) class. | Especially informative for imbalanced datasets; complements AUROC. | (Vela Moreno et al. 2025) |

**Table S2: Performance Evaluation Metrics**

**Table S3. Key Feature Selection Methods Applied in Endometrial Cancer, Outlining Their Advantages, Limitations, and Applications**

| **Algorithm** | **Advantages** | **Disadvantages** | **Application** | **References** |
| --- | --- | --- | --- | --- |
| **Boruta** | Shadow-feature filtering handles noise and missing values | Computationally intensive; parameter-sensitive; redundant feature retention | Retained 18 features after 1000 iterations (PCD-DEGs) | (Kursa and Rudnicki 2010; Pan et al. 2025) |
| **Random Forest** | Compatible with high-dimensionality + non-linear modelling, and low overfitting | Low interpretability; biased importance; tuning required | Radiomics classification: Achieved an AUC ~0.97 | (Ho 1995; Santoro et al. 2024) |
| **Recursive Feature Elimination (RFE)** | Iterative feature pruning; estimator flexible | Multicollinearity sensitive; may drop synergistic features | Narrowed down 4 candidate genes; highlighted ENPP2 as PCOS- EC | (Guyon et al. 2002; Zhang et al. 2024) |
| **LASSO** | Sparse regularization; embedded feature selection | λ dependent; may exclude correlated predictors; linear assumption | Identified 29 prognostic lncRNAs | (Tibshirani 1996; Li et al. 2023). |

**Table S4. Tools for Multi-Omics Data Integration in Machine Learning**

| **Name** | **Core methodology** | **Programming language** | **Custom data support** | **Missing data handling** | **References** |
| --- | --- | --- | --- | --- | --- |
| MOFA2 / MOFA | Matrix factorization framework | R | Yes | Yes | (Muthamilsevan and Palaniappan 2025) |
| iCluster / iClusterPlus / iClusterBayes | Bayesian latent variable model | R | Yes | No | (Chalise et al. 2023) |
| Similarity Network Fusion (SNF) | Network fusion approach | R /MATLAB | Yes | No | (Zhang et al. 2022a) |
| DeepCCA | DL combined with canonical correlation | Python | No | No | (Wróbel et al. 2024) |
| DIABLO | Multi-block CCA / LDA | R | Yes | Yes | (Singh et al. 2019; Morabito et al. 2025) |
| MOLI | DL pipeline for drug response | Python | No | No | (Sharifi-Noghabi et al. 2019; Lee et al. 2022) |
| sCCA | Sparse canonical correlation analysis | R | No | No | (Wróbel et al. 2024) |
| HNMDRP | Network-based drug response prediction | R / MATLAB | No | No | (Zhang et al. 2018; Peng et al. 2022) |
| moCluster | Bayesian clustering model | R | Yes | No | (Subramanian et al. 2020) |

***Supplementary References***

Bruno V, Betti M, D’Ambrosio L, et al (2023) Machine learning endometrial cancer risk prediction model: integrating guidelines of European Society for Medical Oncology with the tumor immune framework. International Journal of Gynecological Cancer 33:1708–1714. https://doi.org/10.1136/IJGC-2023-004671

Chalise P, Kwon D, Fridley BL, Mo Q (2023) Statistical Methods for Integrative Clustering of Multi-omics Data. Methods Mol Biol 2629:73. https://doi.org/10.1007/978-1-0716-2986-4_5

Chen X, Wang Y, Shen M, et al (2020) Deep learning for the determination of myometrial invasion depth and automatic lesion identification in endometrial cancer MR imaging: a preliminary study in a single institution. Eur Radiol 30:4985–4994. https://doi.org/10.1007/S00330-020-06870-1

Goyal M, Tafe LJ, Feng JX, et al (2024) Deep Learning for Grading Endometrial Cancer. Am J Pathol 194:1701. https://doi.org/10.1016/J.AJPATH.2024.05.003

Guan X, Cao R, Liu L, et al (2025) Integrating multi-omics data to optimize immunotherapy in endometrial cancer: a comprehensive study. Discover Oncology 16:1161. https://doi.org/10.1007/S12672-025-02978-2

Guyon I, Weston J, Barnhill S, Vapnik V (2002) Gene selection for cancer classification using support vector machines. Mach Learn 46:389–422. https://doi.org/10.1023/A:1012487302797

Ho TK (1995) Random decision forests. Proceedings of the International Conference on Document Analysis and Recognition, ICDAR 1:278–282. https://doi.org/10.1109/ICDAR.1995.598994

Huang LJ, Liu C, Chen L, et al (2025a) Evaluation of pyroptosis-associated genes in endometrial cancer utilizing a 101-combination machine learning framework and multi-omics data. Front Med (Lausanne) 12:1590405. https://doi.org/10.3389/FMED.2025.1590405

Huang X, Li A, Xu D (2025b) Expression Characteristics and Prognostic Value of KLRG2 in Endometrial Cancer: A Comprehensive Analysis Based on Multi-Omics Data. Biomedicines 13:1592. https://doi.org/10.3390/BIOMEDICINES13071592

Kursa MB, Rudnicki WR (2010) Feature Selection with the Boruta Package. J Stat Softw 36:1–13. https://doi.org/10.18637/JSS.V036.I11

Lee M, Kim PJ, Joe H, Kim HG (2022) Gene-centric multi-omics integration with convolutional encoders for cancer drug response prediction. Comput Biol Med 151:106192. https://doi.org/10.1016/J.COMPBIOMED.2022.106192

Li B, Li X, Ma M, et al (2023) Analysis of long non-coding RNAs associated with disulfidptosis for prognostic signature and immunotherapy response in uterine corpus endometrial carcinoma. Sci Rep 13:22220. https://doi.org/10.1038/S41598-023-49750-6

Liu X, Wang W, Zhang X, et al (2024) Metabolism pathway-based subtyping in endometrial cancer: An integrated study by multi-omics analysis and machine learning algorithms. Mol Ther Nucleic Acids 35. <https://doi.org/10.1016/J.OMTN.2024.102155>

Miller C, Portlock T, Nyaga DM, O’Sullivan JM (2024) A review of model evaluation metrics for machine learning in genetics and genomics. Frontiers in Bioinformatics 4:1457619. https://doi.org/10.3389/FBINF.2024.1457619

Mo Q, Shen R (2023) iClusterPlus: integrative clustering of multiple genomic data sets

Morabito A, De Simone G, Pastorelli R, et al (2025) Algorithms and tools for data-driven omics integration to achieve multilayer biological insights: a narrative review. J Transl Med 23:1–26. https://doi.org/10.1186/S12967-025-06446-X

Muthamilsevan S, Palaniappan A (2025) Integrative multi-omics of gynecological tumors identifies novel singular biomarkers of disease progression. medRxiv [Preprint]. 2025.02.21.25322653. <https://doi.org/10.1101/2025.02.21.25322653>

Pan W, Cheng J, Lin S, et al (2025) Construction of a prognostic model for endometrial cancer related to programmed cell death using WGCNA and machine learning algorithms. Front Immunol 16:1564407. https://doi.org/10.3389/FIMMU.2025.1564407

Peng W, Liu H, Dai W, et al (2022) Predicting cancer drug response using parallel heterogeneous graph convolutional networks with neighborhood interactions. Bioinformatics 38:4546–4553. https://doi.org/10.1093/BIOINFORMATICS/BTAC574

Santoro M, Zybin V, Coada CA, et al (2024) Machine Learning Applied to Pre-Operative Computed-Tomography-Based Radiomic Features Can Accurately Differentiate Uterine Leiomyoma from Leiomyosarcoma: A Pilot Study. Cancers (Basel) 16:1570. https://doi.org/10.3390/CANCERS16081570

Sharifi-Noghabi H, Zolotareva O, Collins CC, Ester M (2019) MOLI: multi-omics late integration with deep neural networks for drug response prediction. Bioinformatics 35:i501. https://doi.org/10.1093/BIOINFORMATICS/BTZ318

Singh A, Shannon CP, Gautier B, et al (2019) DIABLO: an integrative approach for identifying key molecular drivers from multi-omics assays. Bioinformatics 35:3055–3062. https://doi.org/10.1093/BIOINFORMATICS/BTY1054

Song J, Ui A, Mizuguchi K, Watanabe R (2025) A novel method for endometrial cancer patient stratification considering ARID1A protein expression and activity with effective use of multi-omics data. Comput Struct Biotechnol J 27:2614–2625. https://doi.org/10.1016/j.csbj.2025.06.015

Subramanian I, Verma S, Kumar S, et al (2020) Multi-omics Data Integration, Interpretation, and Its Application. Bioinform Biol Insights 14:1177932219899051. https://doi.org/10.1177/1177932219899051

Tibshirani R (1996) Regression Shrinkage and Selection Via the Lasso. J R Stat Soc Series B Stat Methodol 58:267–288. https://doi.org/10.1111/J.2517-6161.1996.TB02080.X

Vela Moreno S, Khatun M, Pasanen A, et al (2025) Explainable machine learning for preoperative relapse prediction in molecularly stratified endometrial cancer: A single-center finnish cohort study. Comput Struct Biotechnol J 31:143. https://doi.org/10.1016/J.CSBJ.2025.12.018

Wróbel S, Turek C, Stępień E, Piwowar M (2024b) Data integration through canonical correlation analysis and its application to OMICs research. J Biomed Inform 151:104575. https://doi.org/10.1016/J.JBI.2023.104575

Xiong X, Zeng L, Zeng F, et al (2025) Bioinformatics exploration of the S1PR1 receptor in various human cancers and its clinical relevance. Discover Oncology 16. https://doi.org/10.1007/S12672-025-02241-8

Zhang F, Wang M, Xi J, et al (2018) A novel heterogeneous network-based method for drug response prediction in cancer cell lines. Sci Rep 8:1–9. https://doi.org/10.1038/S41598-018-21622-4

Zhang G, Peng Z, Yan C, et al (2022a) A novel liver cancer diagnosis method based on patient similarity network and DenseGCN. Sci Rep 12:1–10. https://doi.org/10.1038/S41598-022-10441-3

Zhang Q, Xia T, Qi C, et al (2022b) High expression of S100A2 predicts poor prognosis in patients with endometrial carcinoma. BMC Cancer 22. https://doi.org/10.1186/S12885-022-09180-5,

Zhang X, Liu J, Bai C, et al (2024) Exploring the potential role of ENPP2 in polycystic ovary syndrome and endometrial cancer through bioinformatic analysis. PeerJ 12. https://doi.org/10.7717/PEERJ.18666
